# Publisher Correction: Autophagy facilitates adaptation of budding yeast to respiratory growth by recycling serine for one-carbon metabolism

**DOI:** 10.1038/s41467-020-20648-5

**Published:** 2021-01-04

**Authors:** Alexander I. May, Mark Prescott, Yoshinori Ohsumi

**Affiliations:** 1 Cell Biology Center, Institute of Innovative Research, Tokyo Institute of Technology, 4259-S2-12 Nagatsuta-cho, Midori-ku, Yokohama, 226-8503 Japan; 2 Tokyo Tech World Research Hub Initiative (WRHI), Institute of Innovative Research, Tokyo Institute of Technology, Yokohama, Japan; 3 Monash Biomedicine Discovery Institute, Department of Biochemistry and Molecular Biology, Monash University, Clayton campus, VIC 3800 Australia

**Keywords:** Autophagy, Cell growth, Mitochondria

Correction to: *Nature Communications* 10.1038/s41467-020-18805-x, published online 7 October 2020.

In the original version of this Article, the Supplementary Information file was missing the Supplementary Tables 1–5. The error has now been fixed and the Supplementary Information PDF contains the Supplementary Tables and is available to download from the HTML version of the Article.

## Supplementary information

Supplementary information

