# Supplementary material for: Autophagy facilitates adaptation of budding yeast to respiratory growth by recycling serine for one-carbon metabolism
Source: Nat Commun. 2020 Oct 7;11:5052. doi: 10.1038/s41467-020-18805-x (PMC7542147; doi:10.1038/s41467-020-18805-x)
Supplement: Supplementary file 1 — Supplementary Information [file 41467_2020_18805_MOESM1_ESM.pdf]

Supplementary Information for:

May, A.I., Prescott, M. and Ohsumi, Y.,

Autophagy facilitates adaptation of budding yeast to respiratory growth by recycling serine for one-carbon metabolism

This document contains:

Supplementary Figures 1 - 12
Supplementary Table 1: Antibodies used in this study
Supplementary Table 2: Reagents used in this study
Supplementary Table 3: Strains used in this study
Supplementary Table 4: Plasmids used in this study
Supplementary Table 5: Oligonucleotides used in this study

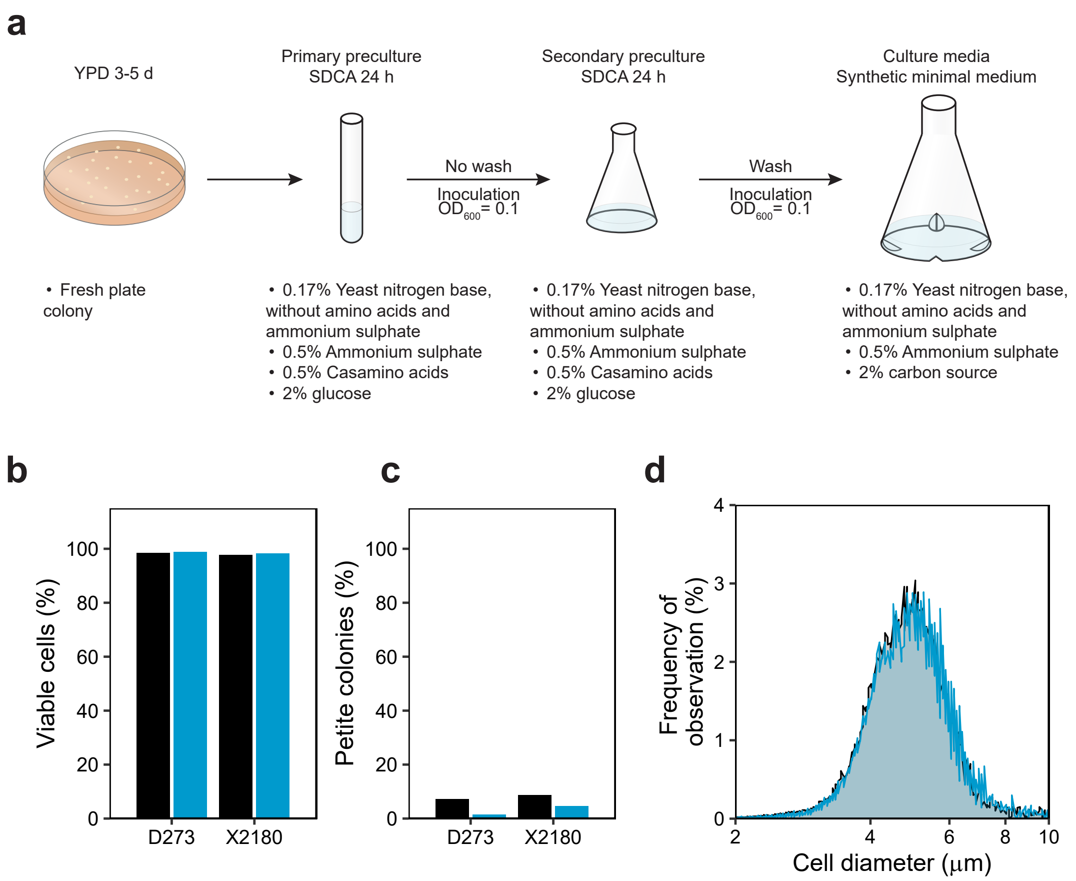

Supplementary Figure 1. Overview of the culture regime employed in this study. (a) Cells were taken from fresh (no older than 5-day-old) YPD plates and inoculated into SDCA medium. Following 24 h of culture, cells were diluted to OD_{600} = 0.1 in fresh SDCA media. After a second 24 h incubation in SDCA, cells were washed in and inoculated to subsequent culture media at OD_{600} = 0.1. (b) Viability and (c) petite frequency of wild-type (black) and *atg2*Δ (blue) cells at the point of inoculation to culture media. (d) Histogram of cell size of wild-type (black) and *atg2*Δ (blue) cells at the point of inoculation to culture media. Error bars = 1 standard deviation. Data are from one (b, d) or two (c) independent experiments.

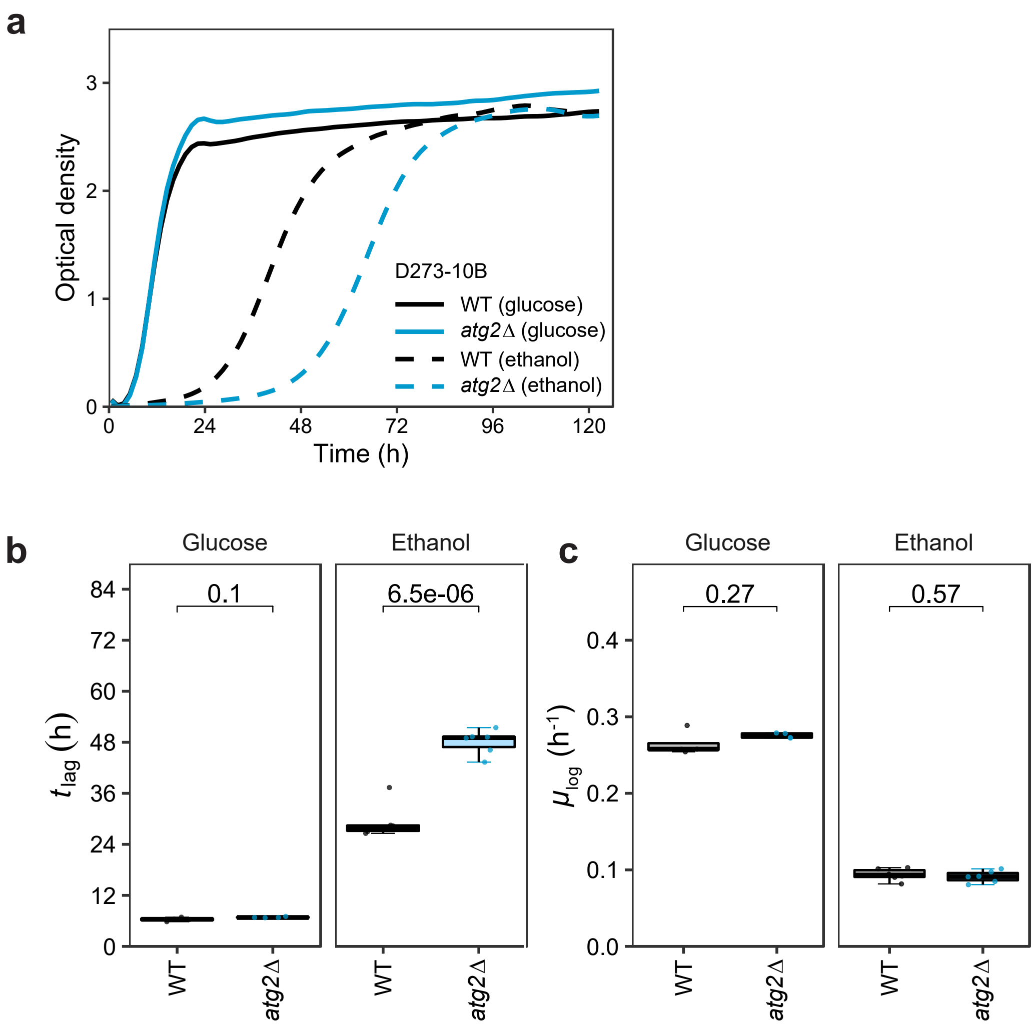

Supplementary Figure 2. Prolonged autophagy mutant respiratory growth t_{lag} is confirmed in an alternative yeast background strain of distinct pedigree. (a) Growth of WT (black lines) and *atg2*Δ (blue lines) strains from the alternative background D273-10B on synthetic media supplemented with glucose (solid lines) or ethanol (broken lines) as carbon sources. Data are a single representative determination of data presented in (b) and (c). (b) Statistical analysis of t_{lag} and (c) μ_{log} for wild-type and *atg2*Δ cells. Data are from at least five independent experiments Mean growth curves from 3 independent determinations of WT (black) and *atg2*Δ (blue) cell growth on a range of fermentative and respiratory carbon sources. Boxplots in (b) and (c) are presented as median (middle bar), 25th and 75th percentiles (upper and lower limits of the box) and 1.5 * interquartile range (whiskers). Sample sizes: n = 4 (glucose) or n = 6 (ethanol). Indicated p-values were calculated using the two-sided Student's t-test with Welch modification. Error bars = 1 standard deviation.

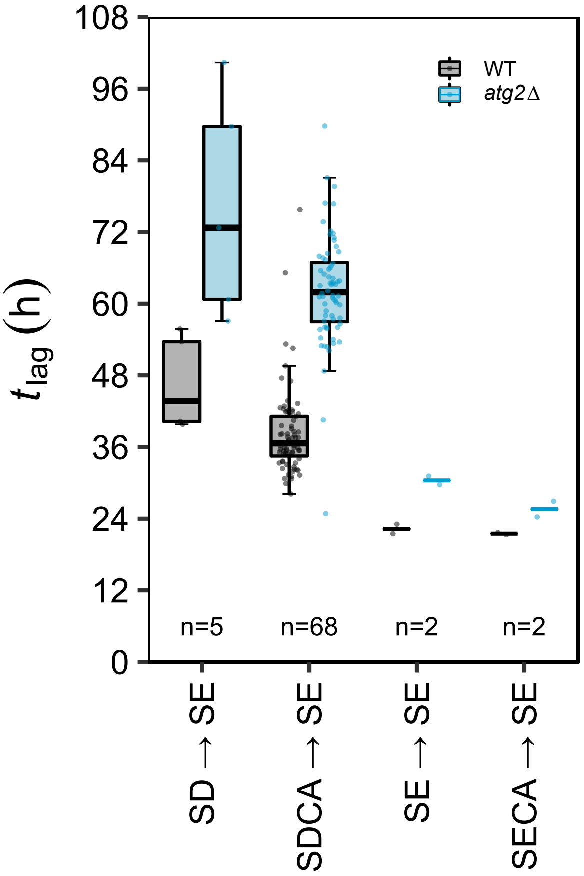

Supplementary Figure 3. The shift from fermentative to non-fermentative media, and not removal of casamino acids, is implicated in the prolonged t_{lag} of *atg2*Δ cells. Shown are boxplots of WT (black) and *atg2*Δ (blue) t_{lag} for cells cultured under the indicated conditions. As n < 3 for cells pre-grown on ethanol media, these data are presented as individual determinations with a bar indicating the mean t_{lag}. SD, synthetic glucose media without casamino acids, SDCA, synthetic glucose media with casamino acids, SE, synthetic ethanol media without casamino acids, SECA, synthetic ethanol media with casamino acids. Data from at least three (n = 3) independent experiments are shown. Error bars = 1 standard deviation. Boxplots are shown as median (middle bar), 25th and 75th percentiles (upper and lower limits of the box) and 1.5 * interquartile range (whiskers).

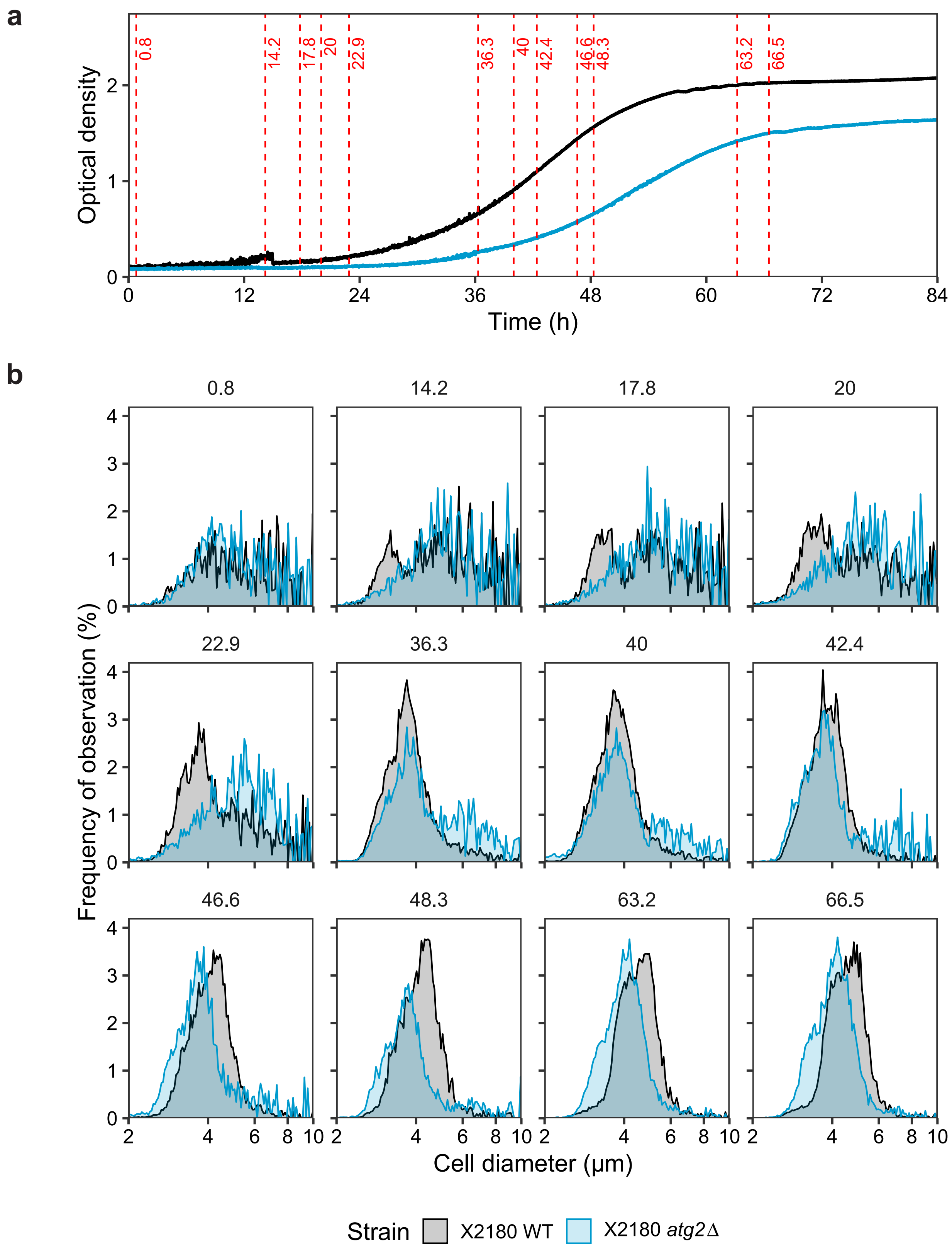

Supplementary Figure 4. Changes in cell size reflect delayed onset of autophagy mutant growth on ethanol media. (a) Growth of WT (black) and *atg2*Δ (blue) cells on ethanol media. Sampling points are indicated by dotted red lines. (b) Cell size was assessed by particle counter at time points indicated in (a) and are presented as histograms of particle size frequency. Black, wild-type cells, blue, *atg2*Δ cells.

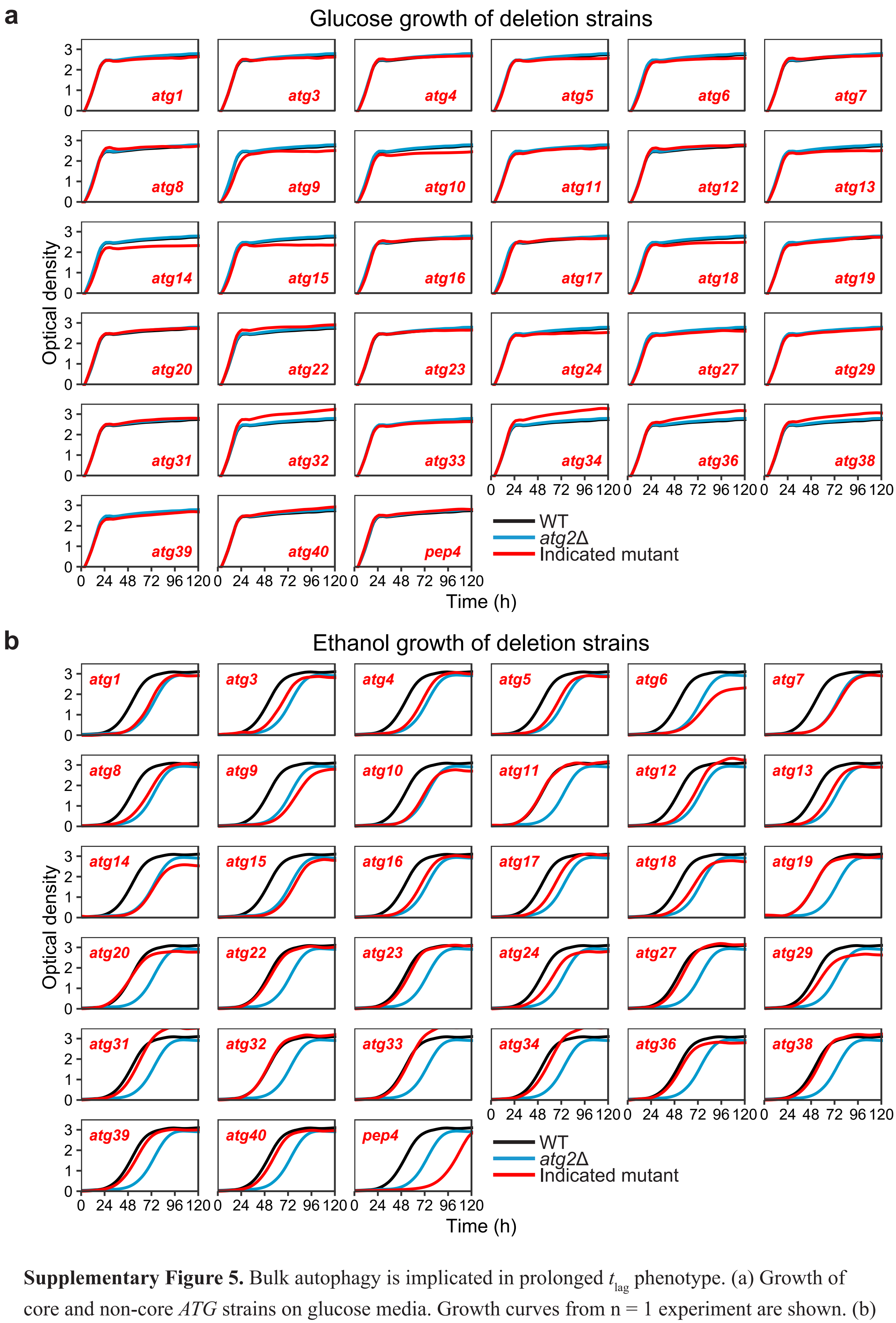

Supplementary Figure 5. Bulk autophagy is implicated in prolonged t_{lag} phenotype. (a) Growth of core and non-core *ATG* strains on glucose media. Growth curves from n = 1 experiment are shown. (b) Growth of core and non-core *ATG* strains on ethanol media. These data were quantified and are shown in Fig. 2. Black lines, wild-type, blue lines, *atg2*Δ, red lines, indicated mutant strain.

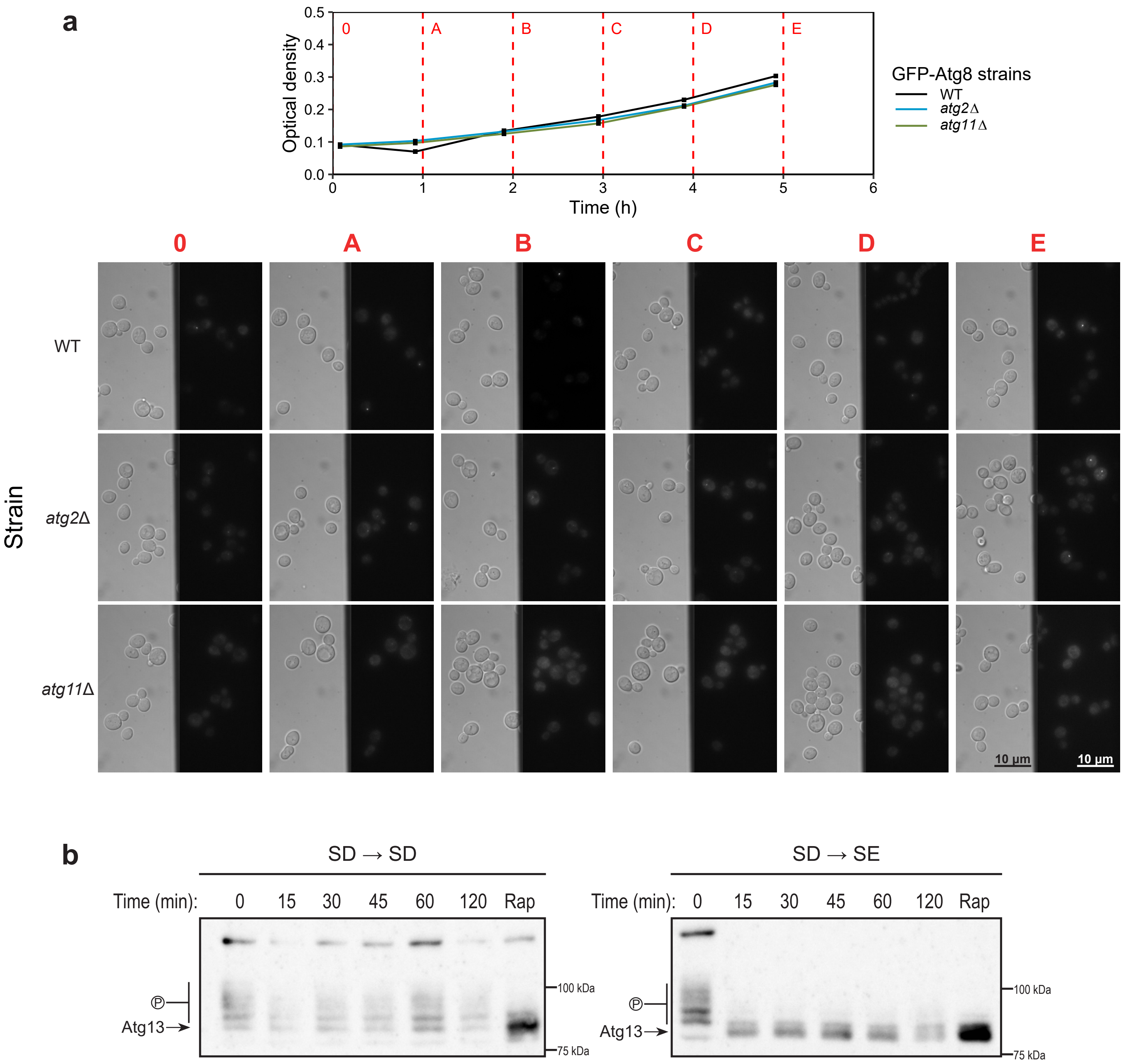

Supplementary Figure 6. Autophagy is not induced during the lag phase of fermentative growth. (a) WT (black), *atg2*Δ (blue) and *atg11*Δ (green) cells expressing GFP-Atg8 were inoculated to SD media under the same conditions as shown in Fig. 3b and observed by microscopy at the indicated time points (broken red lines). Data are from a single experiment and were reproduced twice. (b) The phosphorylation of Atg13 was determined by western blotting in WT cells shifted from synthetic glucose media not containing casamino acids (SD) to SD or synthetic ethanol media not containing casamino acids (SE). Data are from a single experiment and were reproduced three times.

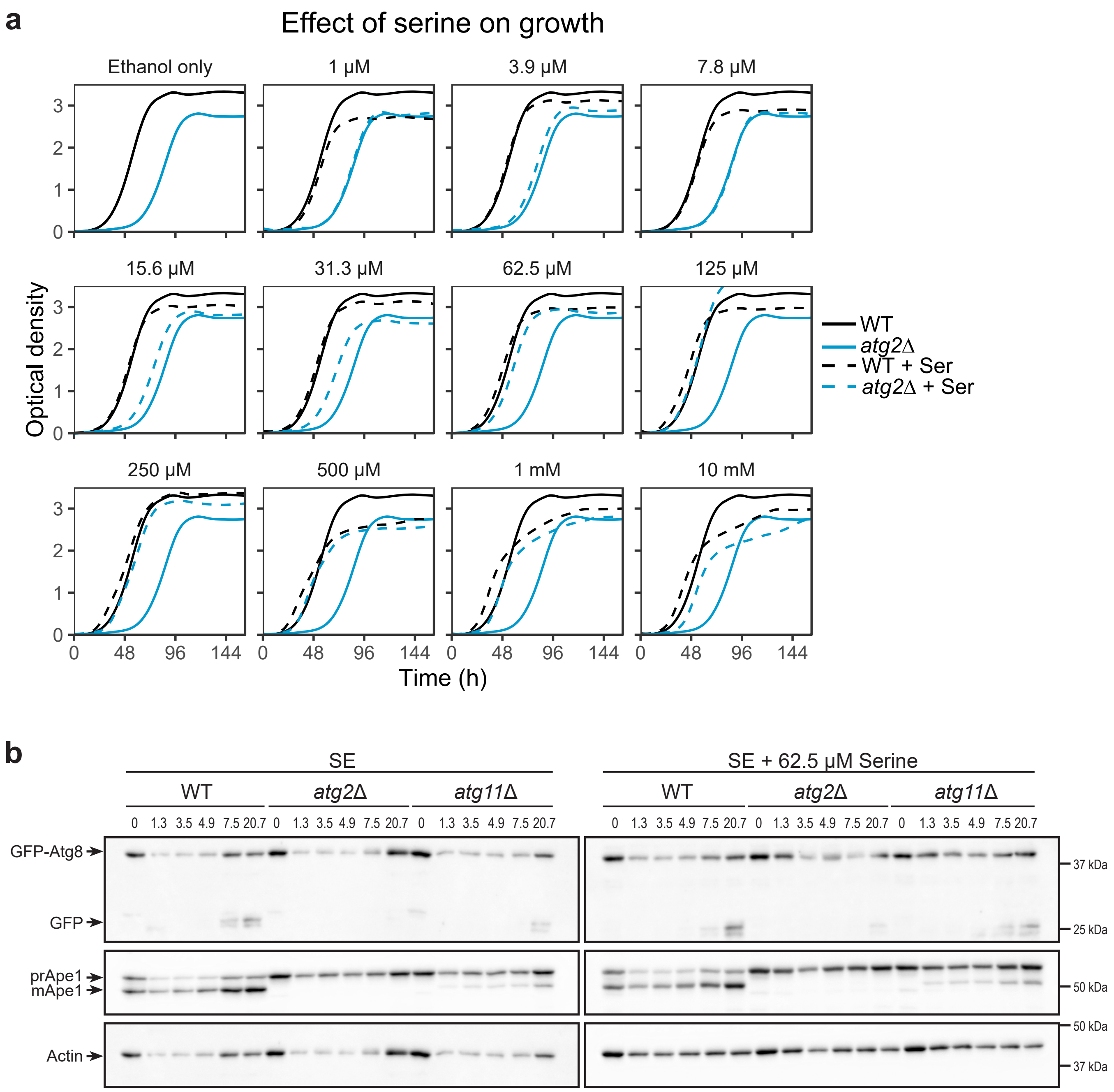

Supplementary Figure 7. Low concentrations of serine are able to alleviate *atg2*Δ t_{lag} but do not affect autophagy induction. (a) Growth curves for WT (black lines) and *atg2*Δ (blue lines) grown on ethanol media alone (solid lines) or with indicated concentrations of serine (broken lines). These data are representative of three independent experiments that were quantified and are shown in Fig. 4. (b) GFP-Atg8 cleavage was assessed in wild-type, *atg2*Δ and *atg11*Δ cells growth on synthetic media with or without serine. Data are from a single experiment.

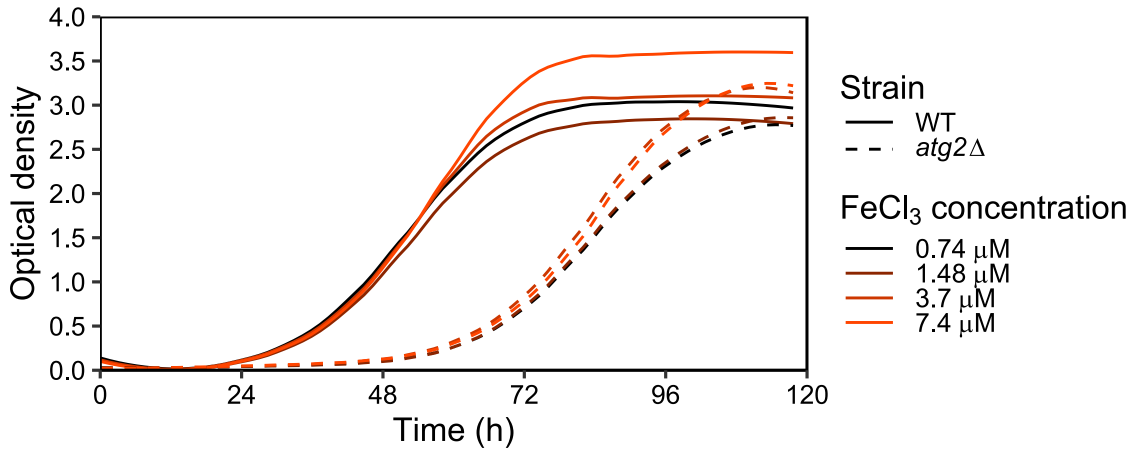

Supplementary Figure 8. Prolonged *atg2*Δ t_{lag} is not alleviated by the supplementation of iron. Ferric chloride was added to respiratory media at the indicated concentrations and WT (solid lines) and *atg2*Δ (broken lines) growth were determined. The average of n = 3 growth experiments is shown.

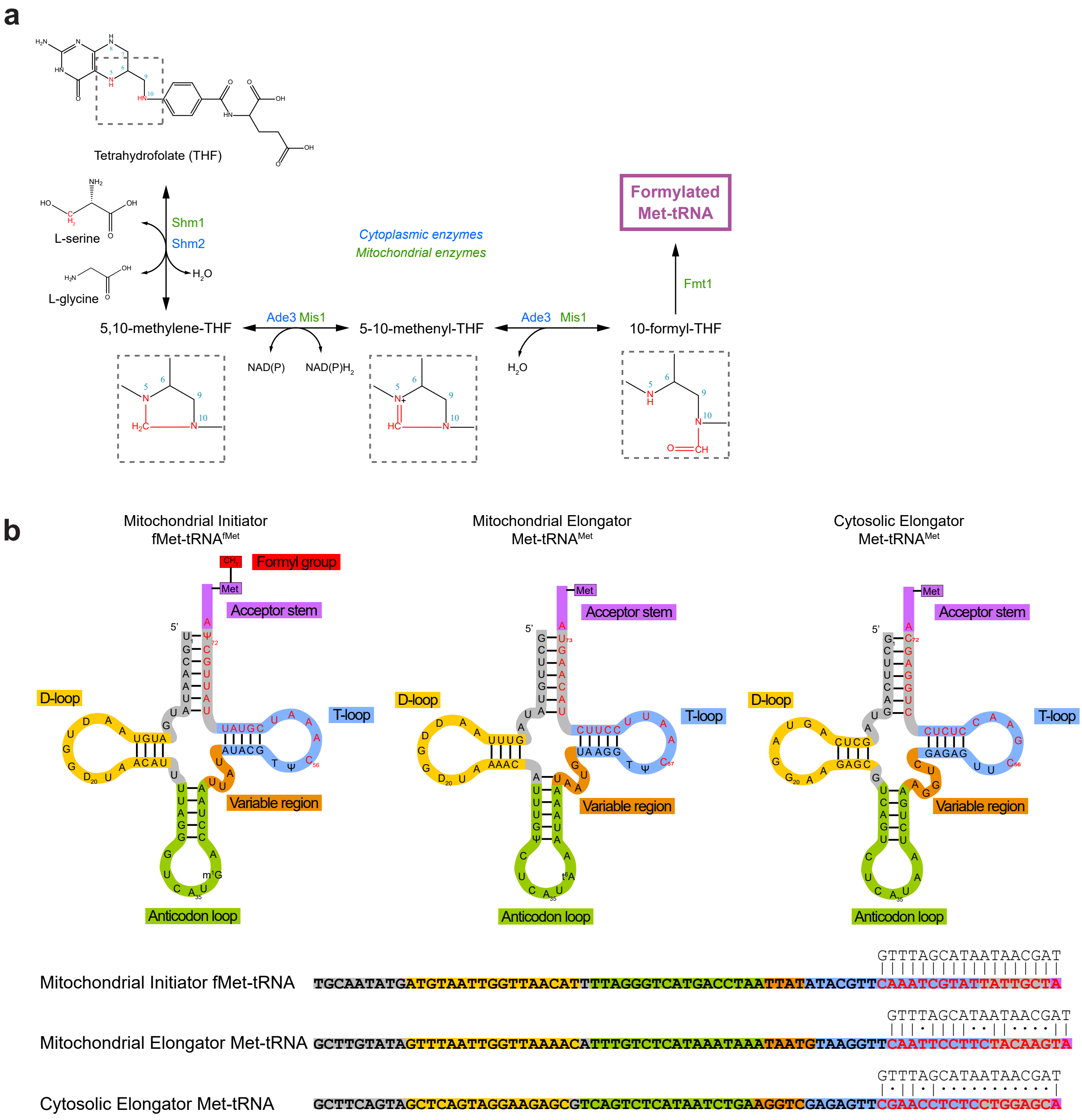

Supplementary Figure 9. Overview of the strategy used to determine formylation of Met-tRNA^{fMet}. (a) detailed overview of the fate of serine in the reactions of one-carbon metabolism. The phenomenon addressed in this paper concerns the mitochondrial reactions (enzymes catalysing these reactions are indicated in green). (b) A comparison of Met-tRNA species found in yeast. The sequence of initiator tRNA in mitochondria is sufficiently different as to allow for its specific probing by northern blotting.

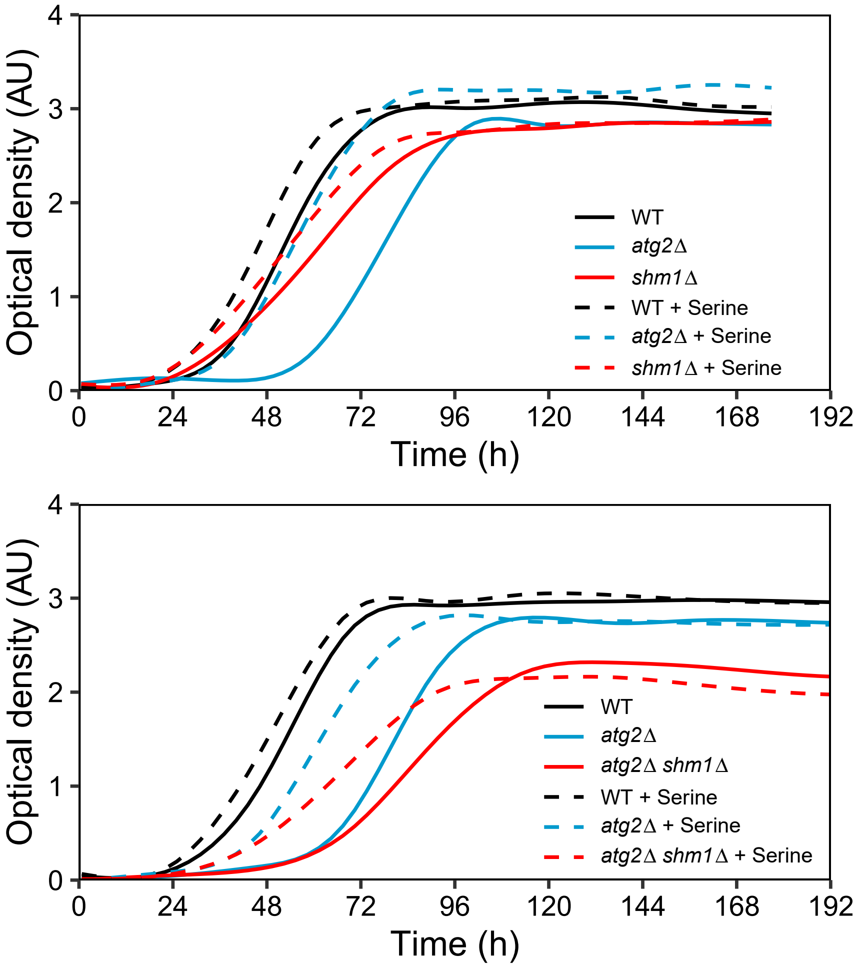

Supplementary Figure 10. The deletion of *SHM1* strongly affects μ_{log} but not the t_{lag} of ethanol-grown cells. WT (black), *atg2*Δ (blue) and *shm1*Δ (red) cells were grown on ethanol media and growth in the absence (solid lines) or presence (broken lines) or serine determined. The average of n = 2 growth experiments is shown.

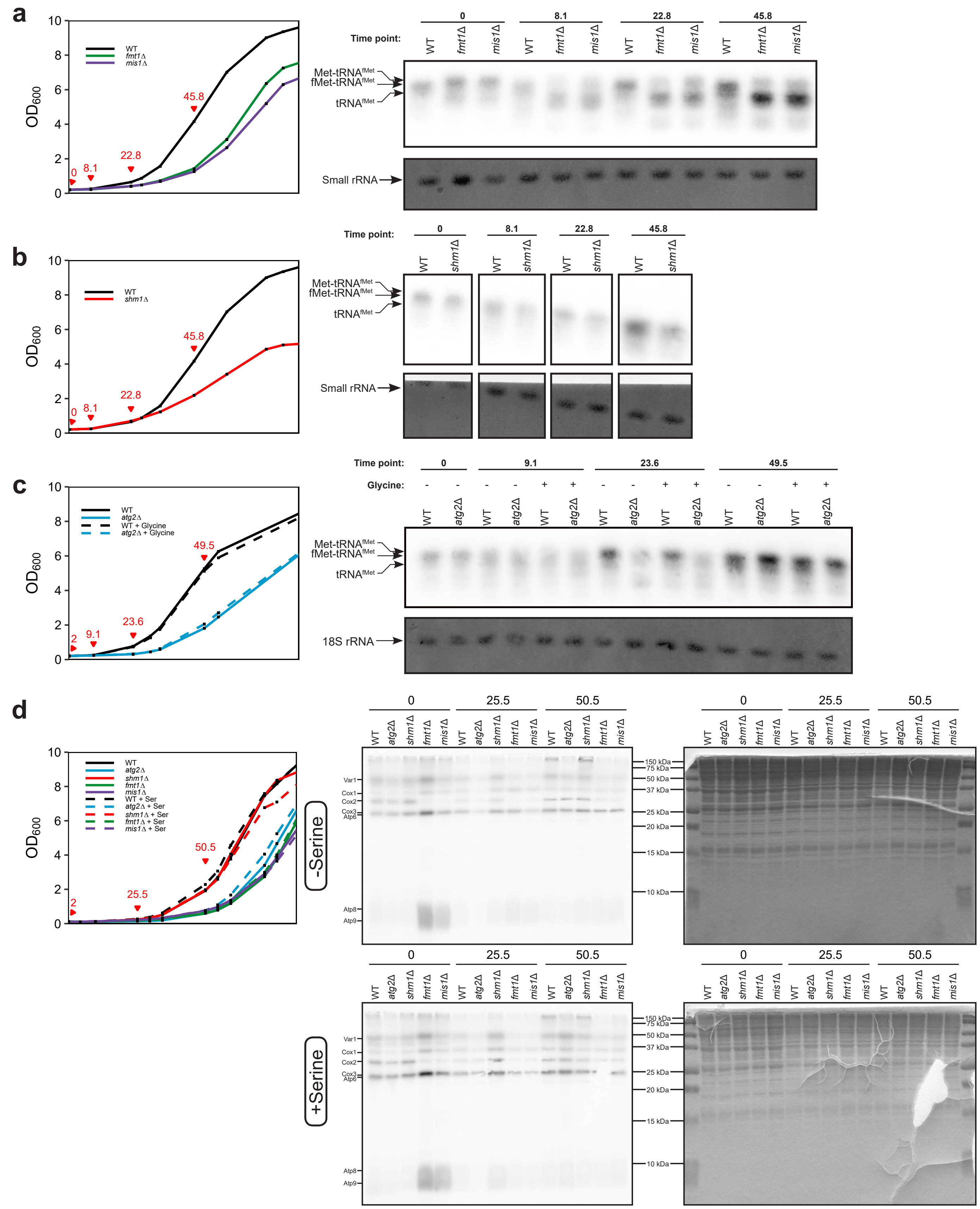

Supplementary Figure 11. t_{lag} duration correlates with initiator tRNA formylation and mitochondrial protein expression in a range of one carbon metabolism mutant strains. (a) Formylation of WT, *fmt1*Δ and *mis1*Δ initiator tRNA under the conditions shown in Fig. 6c. (b) Formylation of WT and *shm1*Δ initiator tRNA under the conditions shown in Fig. 6c. (c) Formylation of WT and *atg2*Δ initiator tRNA under the conditions shown in Fig. 6c, but with glycine supplemented to media instead of serine. (d) Mitochondrial protein expression, as determined by the incorporation of ^{35}S-labelled cysteine and methionine, was determined by autoradiography under the same conditions as shown in Fig. 6d. Samples were collected as indicated in the growth curves at the left of each figure. Data are single determinations from individual experiments.

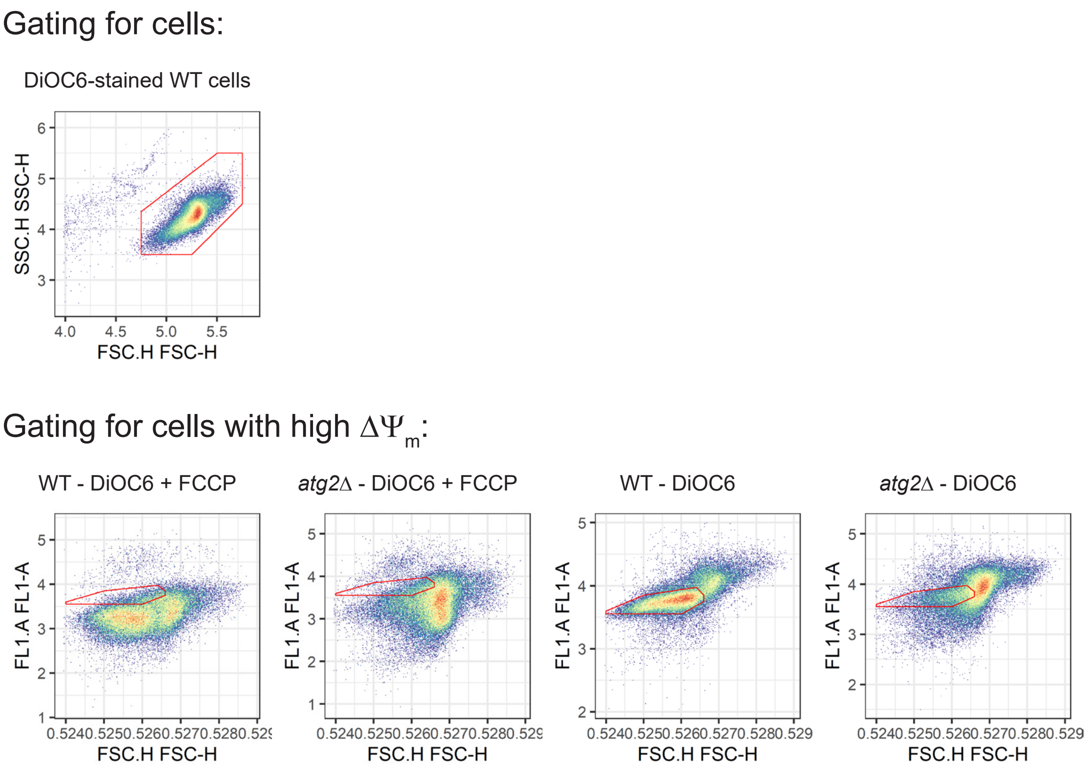

Supplementary Figure 12. Gating strategy used to determine cells with high mitochondrial membrane potential ($\Delta\Psi_m$). First, non-cell debris were removed by gating for cells by forward and side scatter. Following this, the proportion of highly fluorescent newly-emerged cells was identified by gating for the indicated region in the FL1-A channel. The proportion of cells falling within this region are shown in Fig. 5b. The logicle transformation was used for forward and side scatter data. The data shown in this figure are from the t = 26 h time point of the experiment shown in Fig. 5.
