# Supplementary material for: Autophagy facilitates adaptation of budding yeast to respiratory growth by recycling serine for one-carbon metabolism
Source: Nat Commun. 2020 Oct 7;11:5052. doi: 10.1038/s41467-020-18805-x (PMC7542147; doi:10.1038/s41467-020-18805-x)
Supplement: Supplementary file 3 — Reporting Summary [file 41467_2020_18805_MOESM3_ESM.pdf]

nature research

Corresponding author(s): Yoshinori Ohsumi

Last updated by author(s): Aug 14, 2020

Reporting Summary

Nature Research wishes to improve the reproducibility of the work that we publish. This form provides structure for consistency and transparency in reporting. For further information on Nature Research policies, see Authors & Referees and the Editorial Policy Checklist.

Statistics

For all statistical analyses, confirm that the following items are present in the figure legend, table legend, main text, or Methods section.

n/a	Confirmed	
☐	☒	The exact sample size (*n*) for each experimental group/condition, given as a discrete number and unit of measurement
☐	☒	A statement on whether measurements were taken from distinct samples or whether the same sample was measured repeatedly
☐	☒	The statistical test(s) used AND whether they are one- or two-sided<br>*Only common tests should be described solely by name; describe more complex techniques in the Methods section.*
☒	☐	A description of all covariates tested
☒	☐	A description of any assumptions or corrections, such as tests of normality and adjustment for multiple comparisons
☐	☒	A full description of the statistical parameters including central tendency (e.g. means) or other basic estimates (e.g. regression coefficient) AND variation (e.g. standard deviation) or associated estimates of uncertainty (e.g. confidence intervals)
☐	☒	For null hypothesis testing, the test statistic (e.g. *F*, *t*, *r*) with confidence intervals, effect sizes, degrees of freedom and *P* value noted<br>*Give P values as exact values whenever suitable.*
☒	☐	For Bayesian analysis, information on the choice of priors and Markov chain Monte Carlo settings
☒	☐	For hierarchical and complex designs, identification of the appropriate level for tests and full reporting of outcomes
☒	☐	Estimates of effect sizes (e.g. Cohen's *d*, Pearson's *r*), indicating how they were calculated

Our web collection on statistics for biologists contains articles on many of the points above.

Software and code

Policy information about availability of computer code

Data collection	Automated growth data were collected using Advantec TCS062CA communications software (Ver. 100103). Microscopy images were captured using MetaMorph (Ver. 7.7.8.0). Flow cytometry data were collected using BD Accuri C6 software (Ver. 1.0.264.21).
Data analysis	Numerical data analyses were performed using the R programming language (Ver. 3.5.2). For growth data, the Grofit package (Ver 1.1.1-1) was used to estimate growth parameters for statistical analyses. To eliminate measurement noise from automated growth plots, a smoothing function based on loess fitting was applied to raw data using the ggplot2 package (Ver 3.2.1). Note that smoothing was only used on data for presentation, and that all analyses were carried out on raw data. The dose response curve (shown in Fig. 4C) was fitted using the drc package (Ver 3.0-1) in R. Microscopy images were processed using ImageJ (Fiji Ver 1.52p) with no modification of contrast or brightness. Deconvolution was performed using AutoQuant X 3.0.5. Throughout the paper, averages represent mean values, and error bars represent one standard deviation from the mean. Significance was assumed where $p \leq 0.05$. No data were excluded for the purposes of statistical analyses. Specific statistical parameters are indicated in figure legends where relevant.

For manuscripts utilizing custom algorithms or software that are central to the research but not yet described in published literature, software must be made available to editors/reviewers. We strongly encourage code deposition in a community repository (e.g. GitHub). See the Nature Research guidelines for submitting code & software for further information.

Data

Policy information about availability of data

All manuscripts must include a data availability statement. This statement should provide the following information, where applicable:

- Accession codes, unique identifiers, or web links for publicly available datasets
- A list of figures that have associated raw data
- A description of any restrictions on data availability

Growth curves used to produce the data shown in Fig. 2 are shown in Fig. S4. Growth curves used to construct Fig. 4C are shown in Fig. S5a. No restrictions apply to the data collected for this paper, which will be provided upon request.

Field-specific reporting

Please select the one below that is the best fit for your research. If you are not sure, read the appropriate sections before making your selection.

☒ Life sciences ☐ Behavioural & social sciences ☐ Ecological, evolutionary & environmental sciences

For a reference copy of the document with all sections, see nature.com/documents/nr-reporting-summary-flat.pdf

Life sciences study design

All studies must disclose on these points even when the disclosure is negative.

Sample size	Sample sizes were not predetermined for the purposes of statistical analyses. However, statistical analyses were only performed on datasets with at least 3 independent determinations. Sample sizes are reported either with the relevant figures or in the corresponding figure legends.
Data exclusions	No data were excluded for the purposes of statistical analysis.
Replication	Reproducibility of growth data was confirmed through the inclusion of multiple replicates (described above) and verification in an alternative yeast background strain. Some variability in the timing of growth onset was observed in the dataset, but relative trends in growth in response to the investigated conditions were highly reproducible. The variability of data is presented in boxplots in the manuscript. Western blotting data presented in this report are representative of at least two experiments that reproduced the same result. Northern blots presented are representative for two experiments (for Fig. 6) or single determinations (Supplementary Fig. 11). Otherwise, sample sizes are reported in the figure legends or the figure panels with individual data points reported where possible. Results that were not reliably reproduced are not presented in this study. All replicates presented in this report are independent biological replicates (i.e., grown from separate precultures and processed separately).
Randomization	The order of tubes used to grow strains by automated sampler was randomised to account for differences between individual sensors. For microscopy, random fields of view were selected by light microscopy before capturing fluorescence images. Due to the automated or otherwise non-subjective nature of the experimental techniques employed in this study, the experimenter was not blinded to samples, and covariates were not considered relevant in this study due to the use of a microbial experimental model under automated or otherwise controlled experimental conditions.
Blinding	The investigator was blinded to samples for microscopy. Otherwise, blinding was not carried out as the methods used to collect data in this report were automated, not specific to individual samples or not subjective in nature. With the exception of data collection at the microscope, we therefore do not consider blinding relevant in this study.

Reporting for specific materials, systems and methods

We require information from authors about some types of materials, experimental systems and methods used in many studies. Here, indicate whether each material, system or method listed is relevant to your study. If you are not sure if a list item applies to your research, read the appropriate section before selecting a response.

Materials & experimental systems

n/a	Involved in the study	
☐	☒	Antibodies
☒	☐	Eukaryotic cell lines
☒	☐	Palaeontology
☒	☐	Animals and other organisms
☒	☐	Human research participants
☒	☐	Clinical data

Methods

n/a	Involved in the study	
☒	☐	ChIP-seq
☐	☒	Flow cytometry
☒	☐	MRI-based neuroimaging

Antibodies

Antibodies used	Mouse monoclonal Anti-GFP, clones 7.1 and 13.1 (Roche 11814460001), Mouse monoclonal Anti-β-actin (Wako 011-24554), Rabbit polyclonal Anti-Ape1 (Ohsumi lab stock), Rabbit polyclonal Anti-Atg13 (Ohsumi lab stock). Ohsumi lab stock antibodies were previously developed in-house and are available to researchers upon reasonable request.
Validation	Anti-GFP and Anti-β-actin antibodies are widely used antibodies with relevant validation data provided at manufacturer websites. The Anti-Ape1 and Anti-Atg13 antibodies are Ohsumi lab stocks that have been used in numerous publications from our and other labs (e.g. Yamasaki et al., Molecular Cell, 2020 for anti-Ape1 and Adachi et al., Journal of Biological Chemistry, 2017). All antibodies ran at the expected sizes for target proteins. Ape1 processing was observed for the Anti-Ape1 antibody, as expected, and the widely reported phosphorylation of Anti-Atg13 is clearly observed using the Atg13 antibody.

Flow Cytometry

Plots

Confirm that:

- [x] The axis labels state the marker and fluorochrome used (e.g. CD4-FITC).
- [x] The axis scales are clearly visible. Include numbers along axes only for bottom left plot of group (a 'group' is an analysis of identical markers).
- [x] All plots are contour plots with outliers or pseudocolor plots.
- [x] A numerical value for number of cells or percentage (with statistics) is provided.

Methodology

Sample preparation	Cells were removed from culture media, washed once in fresh culture media and suspended in the same media with dye. Samples were agitated in the dark for 15 min and then washed twice in fresh media. Cells were then suspended in fresh media, briefly sonicated and subjected to flow cytometry.
Instrument	BD Accuri C6
Software	BD Accuri C6 software (provided by manufacturer, Ver. 1.0.264.21) and R statistical software.
Cell population abundance	50,000 cells were analysed per sample. Over 95% of these cells were singlets (confirmed by FSC/SSC gating).
Gating strategy	Singlets were first identified by FSC/SSC plotting. Following this, the proportion of cells falling within a peak was gated from a histogram of fluorescence intensities. The gating strategy is shown in Fig. S12.

- [x] Tick this box to confirm that a figure exemplifying the gating strategy is provided in the Supplementary Information.
