# Supplementary material for: Publisher Correction: Autophagy facilitates adaptation of budding yeast to respiratory growth by recycling serine for one-carbon metabolism
Source: Nat Commun. 2021 Jan 4;12:206. doi: 10.1038/s41467-020-20648-5 (PMC7782837; doi:10.1038/s41467-020-20648-5)
Supplement: Supplementary file 1 — Supplementary information [file 41467_2020_20648_MOESM1_ESM.pdf]

Supplementary Information for:

May, A.I., Prescott, M. and Ohsumi, Y.,

Autophagy facilitates adaptation of budding yeast to respiratory growth by recycling serine for one-carbon metabolism

This document contains:

Supplementary Figures 1 - 12
Supplementary Table 1: Antibodies used in this study
Supplementary Table 2: Reagents used in this study
Supplementary Table 3: Strains used in this study
Supplementary Table 4: Plasmids used in this study
Supplementary Table 5: Oligonucleotides used in this study

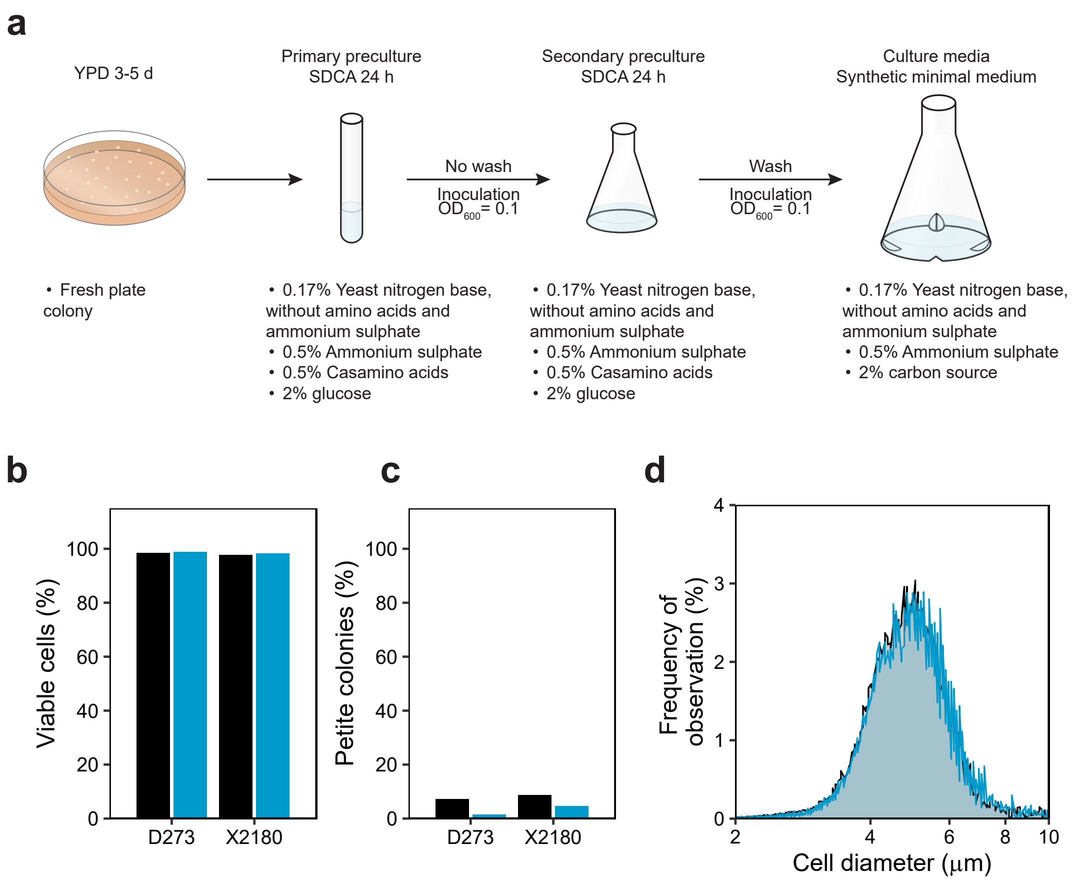

Supplementary Figure 1. Overview of the culture regime employed in this study. (a) Cells were taken from fresh (no older than 5-day-old) YPD plates and inoculated into SDCA medium. Following 24 h of culture, cells were diluted to $OD_{600} = 0.1$ in fresh SDCA media. After a second 24 h incubation in SDCA, cells were washed in and inoculated to subsequent culture media at $OD_{600} = 0.1$. (b) Viability and (c) petite frequency of wild-type (black) and *atg2*Δ (blue) cells at the point of inoculation to culture media. (d) Histogram of cell size of wild-type (black) and *atg2*Δ (blue) cells at the point of inoculation to culture media. Error bars = 1 standard deviation. Data are from one (b, d) or two (c) independent experiments.

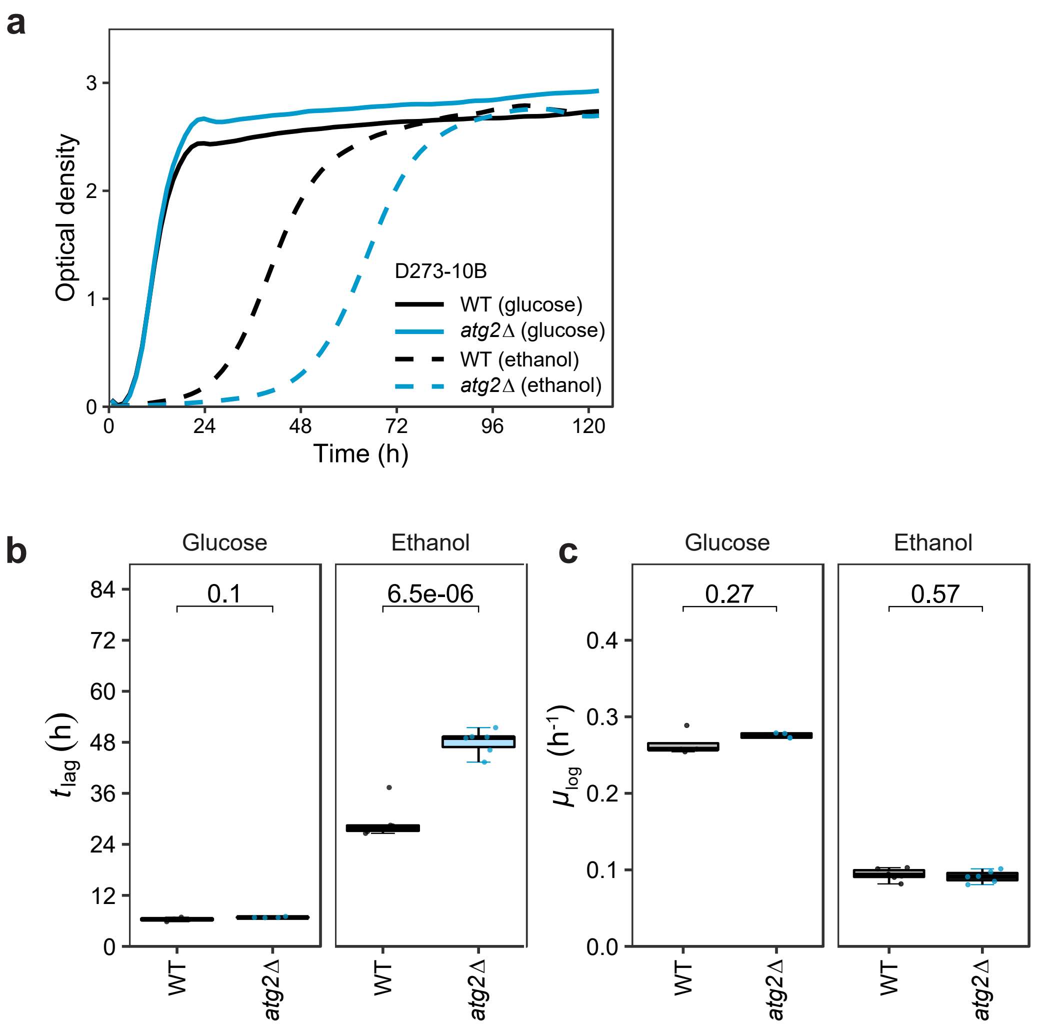

Supplementary Figure 2. Prolonged autophagy mutant respiratory growth t_{lag} is confirmed in an alternative yeast background strain of distinct pedigree. (a) Growth of WT (black lines) and *atg2*Δ (blue lines) strains from the alternative background D273-10B on synthetic media supplemented with glucose (solid lines) or ethanol (broken lines) as carbon sources. Data are a single representative determination of data presented in (b) and (c). (b) Statistical analysis of t_{lag} and (c) μ_{log} for wild-type and *atg2*Δ cells. Data are from at least five independent experiments Mean growth curves from 3 independent determinations of WT (black) and *atg2*Δ (blue) cell growth on a range of fermentative and respiratory carbon sources. Boxplots in (b) and (c) are presented as median (middle bar), 25th and 75th percentiles (upper and lower limits of the box) and 1.5 * interquartile range (whiskers). Sample sizes: n = 4 (glucose) or n = 6 (ethanol). Indicated p-values were calculated using the two-sided Student's t-test with Welch modification. Error bars = 1 standard deviation.

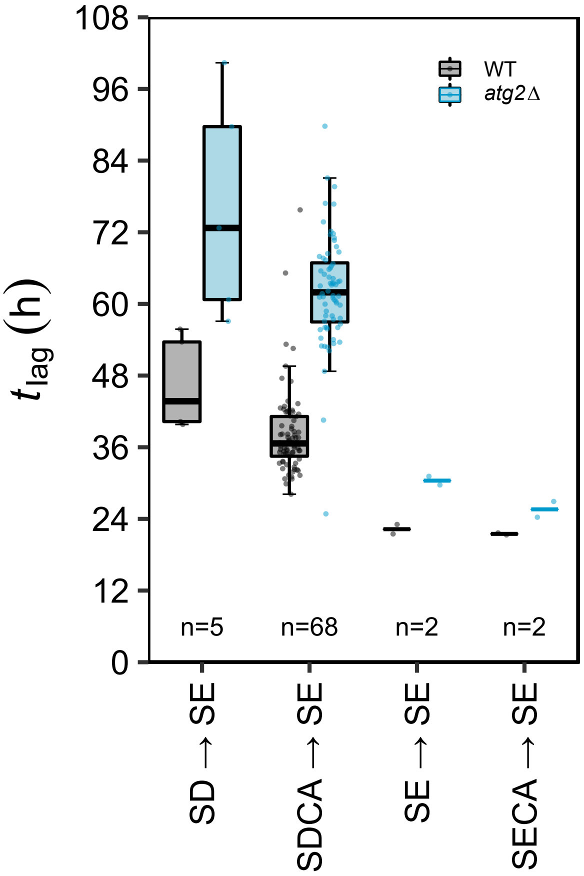

Supplementary Figure 3. The shift from fermentative to non-fermentative media, and not removal of casamino acids, is implicated in the prolonged t_{lag} of *atg2*Δ cells. Shown are boxplots of WT (black) and *atg2*Δ (blue) t_{lag} for cells cultured under the indicated conditions. As n < 3 for cells pre-grown on ethanol media, these data are presented as individual determinations with a bar indicating the mean t_{lag}. SD, synthetic glucose media without casamino acids, SDCA, synthetic glucose media with casamino acids, SE, synthetic ethanol media without casamino acids, SECA, synthetic ethanol media with casamino acids. Data from at least three (n = 3) independent experiments are shown. Error bars = 1 standard deviation. Boxplots are shown as median (middle bar), 25th and 75th percentiles (upper and lower limits of the box) and 1.5 * interquartile range (whiskers).

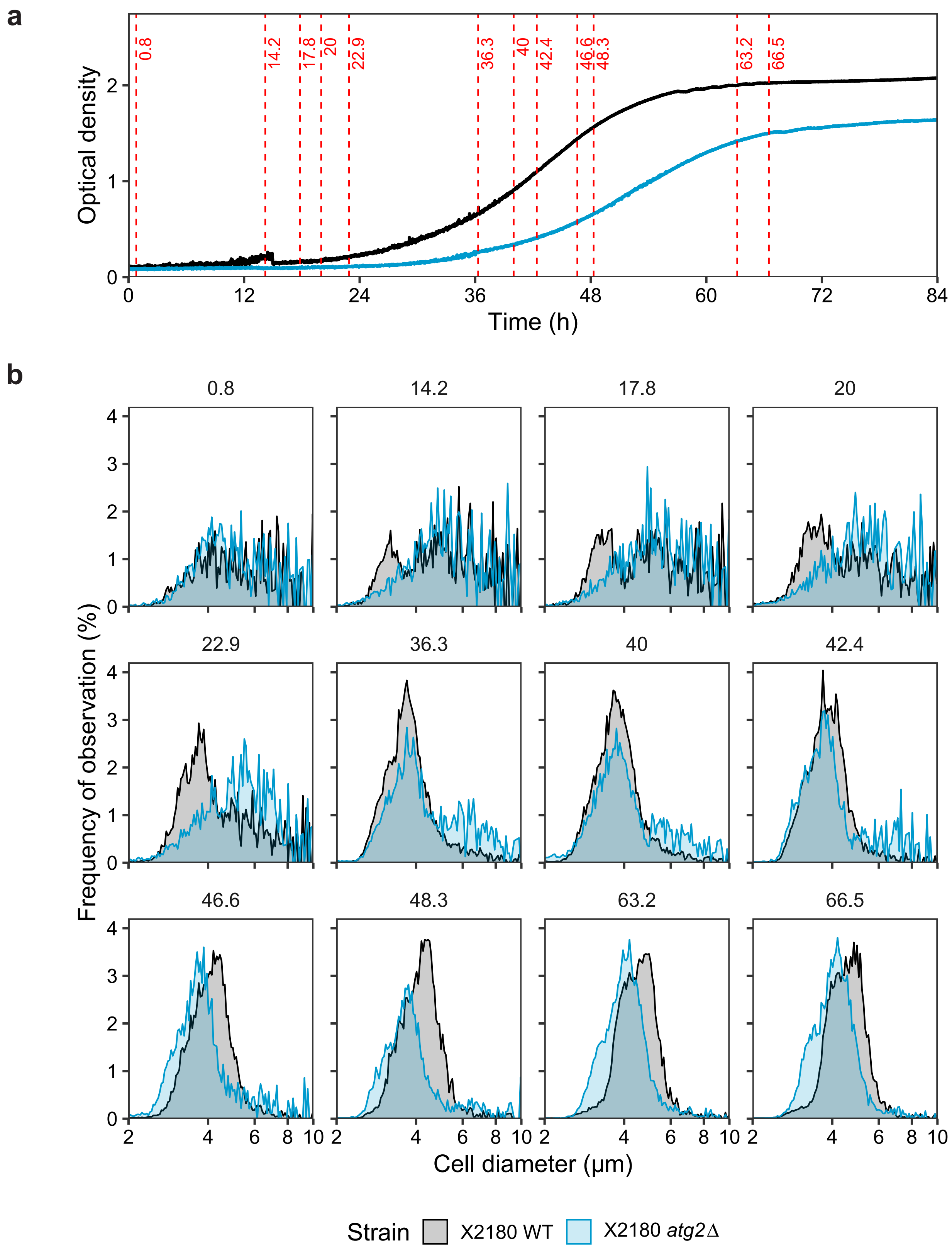

Supplementary Figure 4. Changes in cell size reflect delayed onset of autophagy mutant growth on ethanol media. (a) Growth of WT (black) and *atg2*Δ (blue) cells on ethanol media. Sampling points are indicated by dotted red lines. (b) Cell size was assessed by particle counter at time points indicated in (a) and are presented as histograms of particle size frequency. Black, wild-type cells, blue, *atg2*Δ cells.

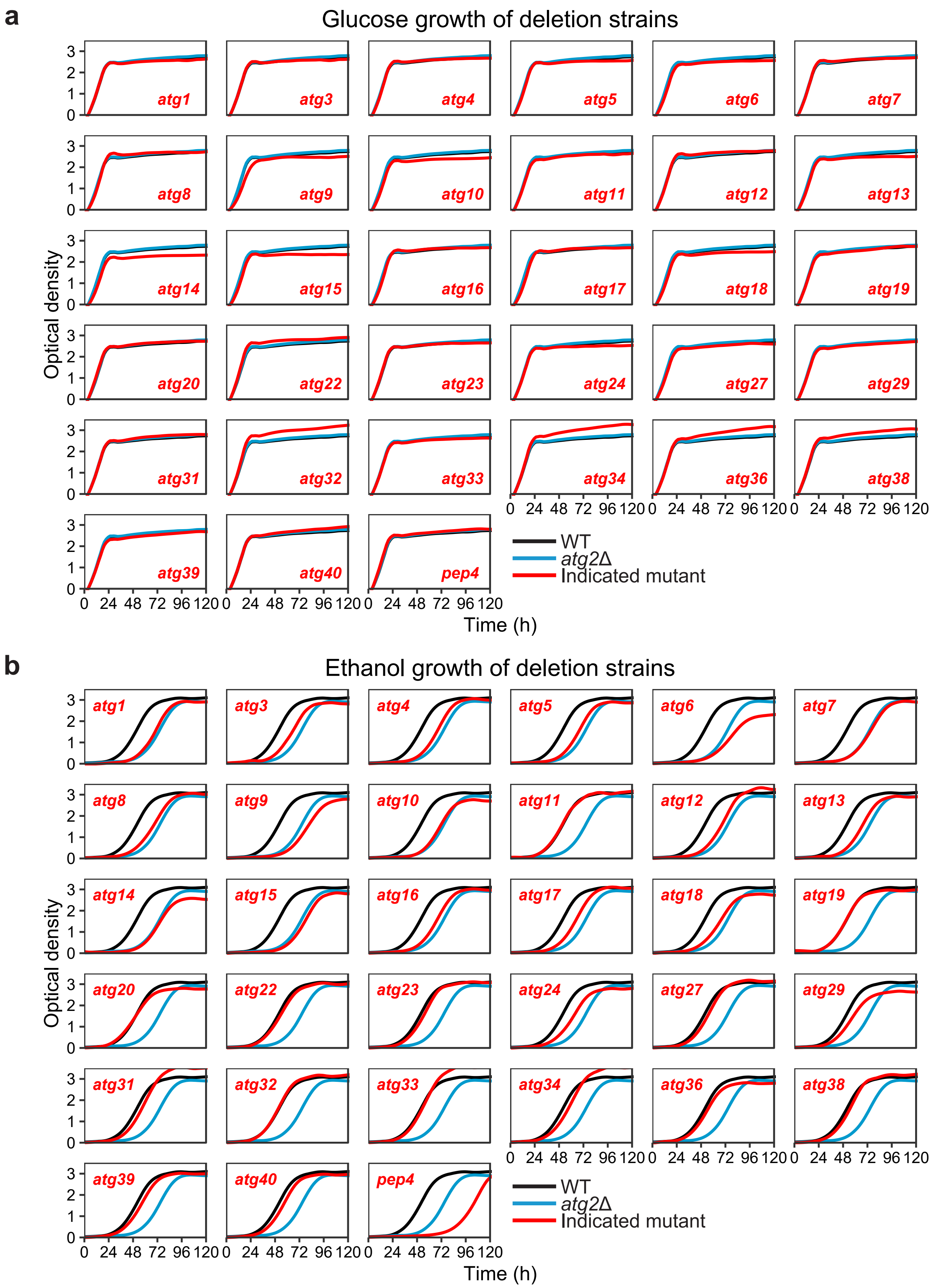

Supplementary Figure 5. Bulk autophagy is implicated in prolonged t_{lag} phenotype. (a) Growth of core and non-core *ATG* strains on glucose media. Growth curves from n = 1 experiment are shown. (b) Growth of core and non-core *ATG* strains on ethanol media. These data were quantified and are shown in Fig. 2. Black lines, wild-type, blue lines, *atg2*Δ, red lines, indicated mutant strain.

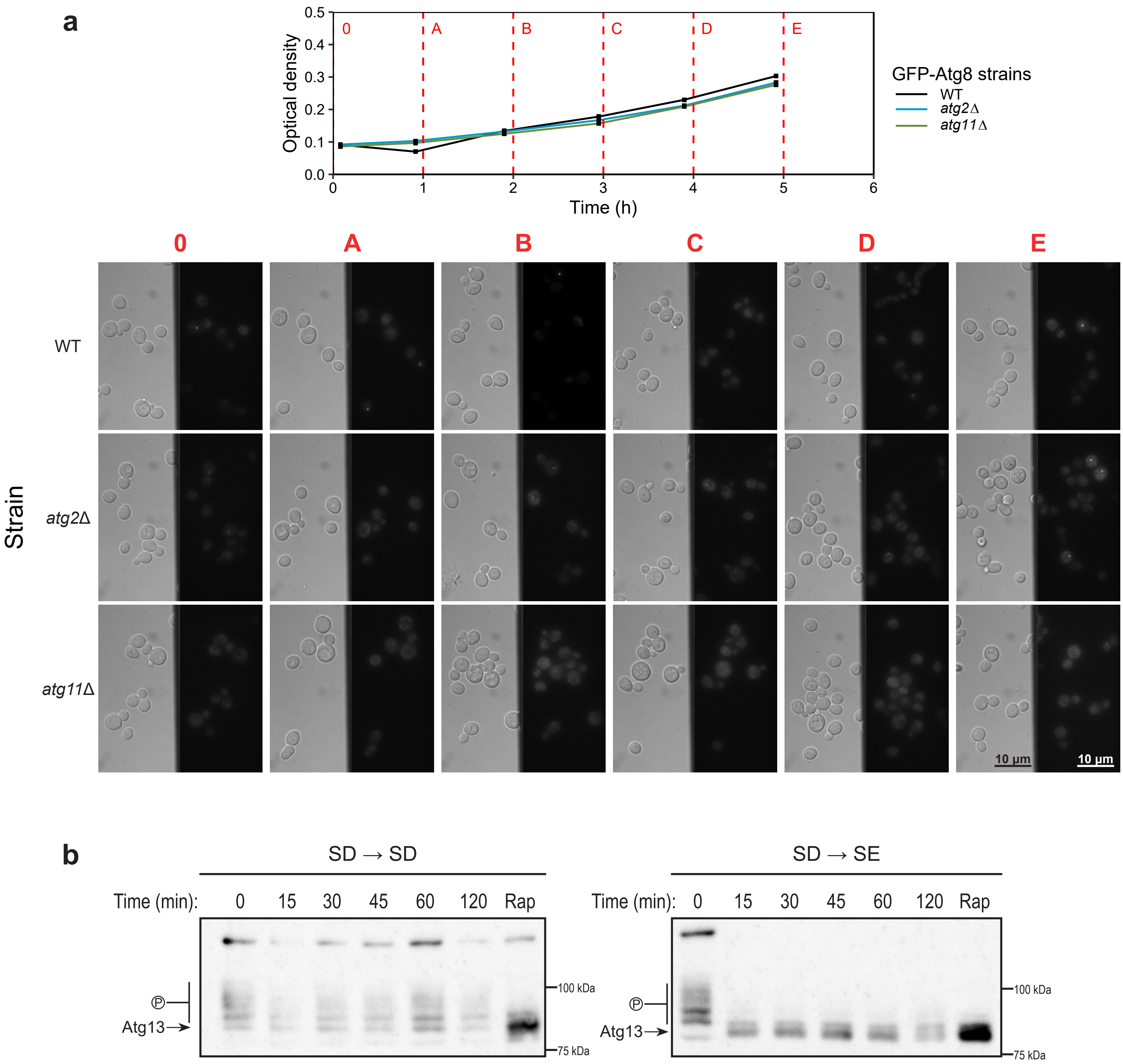

Supplementary Figure 6. Autophagy is not induced during the lag phase of fermentative growth. (a) WT (black), *atg2*Δ (blue) and *atg11*Δ (green) cells expressing GFP-Atg8 were inoculated to SD media under the same conditions as shown in Fig. 3b and observed by microscopy at the indicated time points (broken red lines). Data are from a single experiment and were reproduced twice. (b) The phosphorylation of Atg13 was determined by western blotting in WT cells shifted from synthetic glucose media not containing casamino acids (SD) to SD or synthetic ethanol media not containing casamino acids (SE). Data are from a single experiment and were reproduced three times.

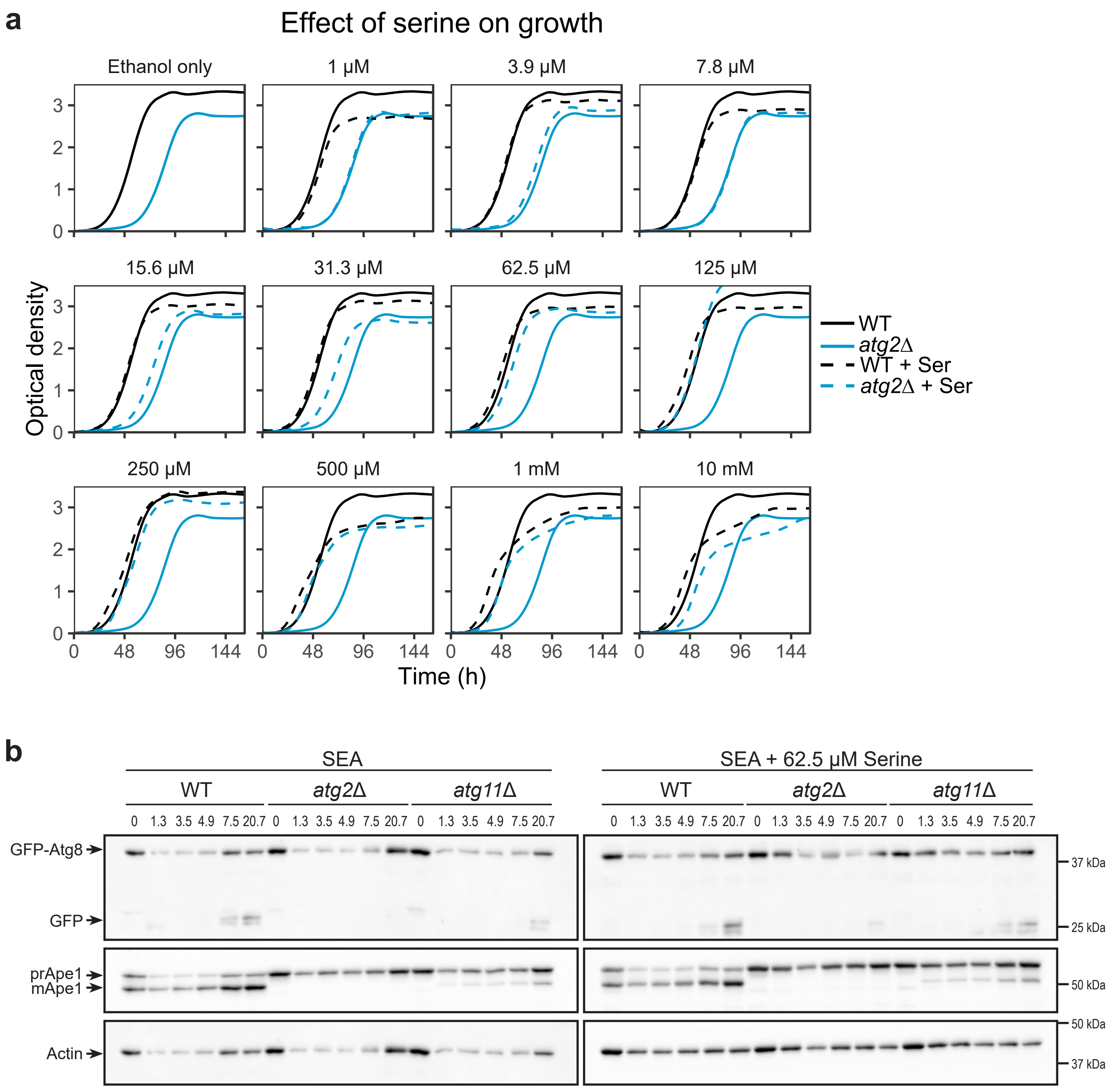

Supplementary Figure 7. Low concentrations of serine are able to alleviate *atg2*Δ t_{lag} but do not affect autophagy induction. (a) Growth curves for WT (black lines) and *atg2*Δ (blue lines) grown on ethanol media alone (solid lines) or with indicated concentrations of serine (broken lines). These data are representative of three independent experiments that were quantified and are shown in Fig. 4. (b) GFP-Atg8 cleavage was assessed in wild-type, *atg2*Δ and *atg11*Δ cells growth on synthetic media with or without serine. Data are from a single experiment.

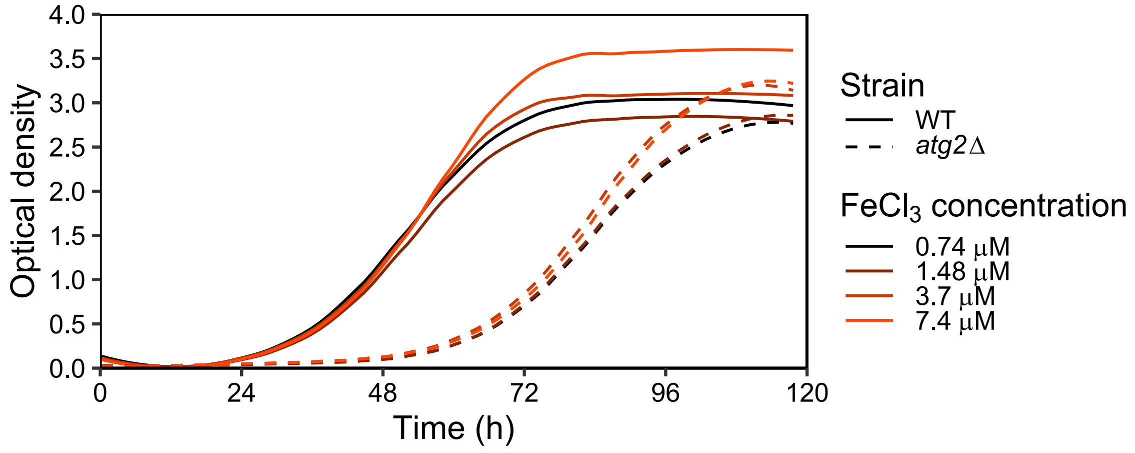

Supplementary Figure 8. Prolonged *atg2*Δ t_{lag} is not alleviated by the supplementation of iron. Ferric chloride was added to respiratory media at the indicated concentrations and WT (solid lines) and *atg2*Δ (broken lines) growth were determined. The average of n = 3 growth experiments is shown.

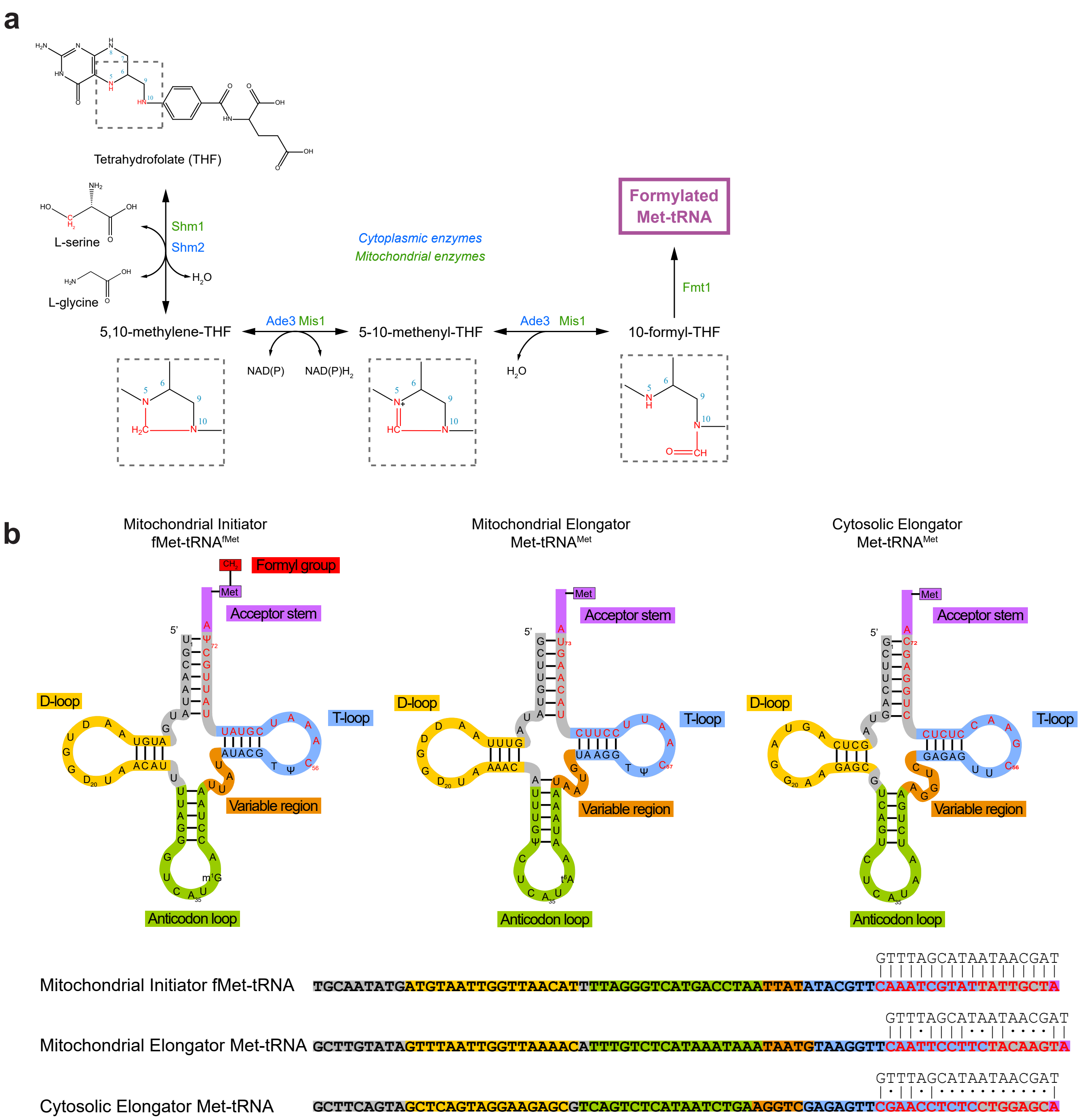

Supplementary Figure 9. Overview of the strategy used to determine formylation of Met-$tRNA^{fMet}$. (a) detailed overview of the fate of serine in the reactions of one-carbon metabolism. The phenomenon addressed in this paper concerns the mitochondrial reactions (enzymes catalysing these reactions are indicated in green). (b) A comparison of Met-tRNA species found in yeast. The sequence of initiator tRNA in mitochondria is sufficiently different as to allow for its specific probing by northern blotting.

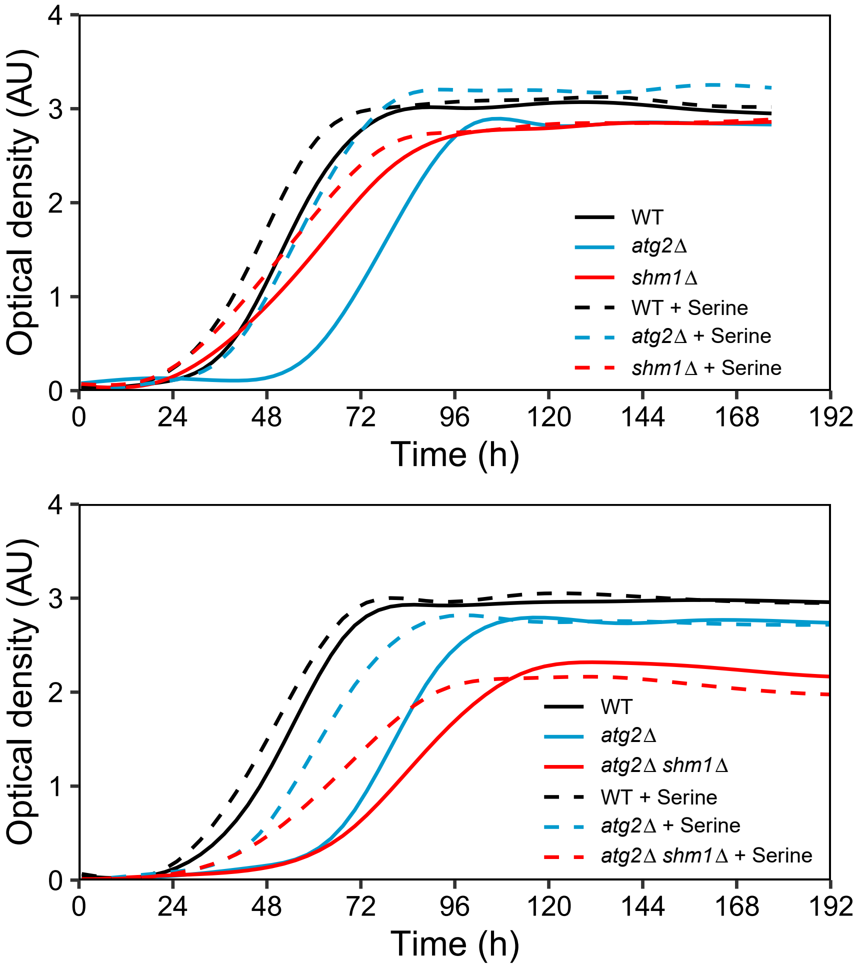

Supplementary Figure 10. The deletion of *SHM1* strongly affects μ_{log} but not the t_{lag} of ethanol-grown cells. WT (black), *atg2*Δ (blue) and *shm1*Δ (red) cells were grown on ethanol media and growth in the absence (solid lines) or presence (broken lines) or serine determined. The average of n = 2 growth experiments is shown.

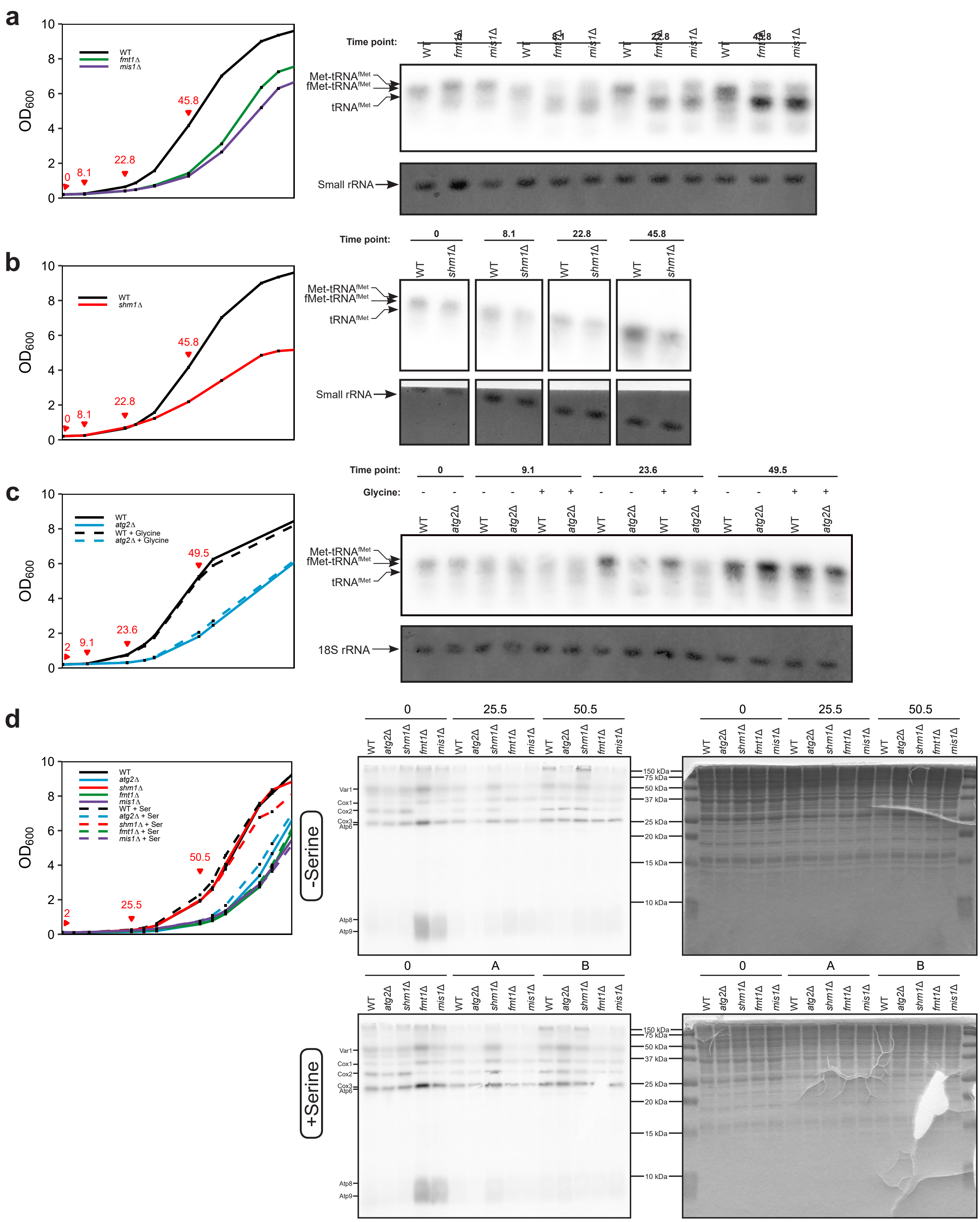

Supplementary Figure 11. t_{lag} duration correlates with initiator tRNA formylation and mitochondrial protein expression in a range of one carbon metabolism mutant strains. (a) Formylation of WT, *fmt1*Δ and *mis1*Δ initiator tRNA under the conditions shown in Fig. 6c. (b) Formylation of WT and *shm1*Δ initiator tRNA under the conditions shown in Fig. 6c. (c) Formylation of WT and *atg2*Δ initiator tRNA under the conditions shown in Fig. 6c, but with glycine supplemented to media instead of serine. (d) Mitochondrial protein expression, as determined by the incorporation of ^{35}S-labelled cysteine and methionine, was determined by autoradiography under the same conditions as shown in Fig. 6d. Samples were collected as indicated in the growth curves at the left of each figure. Data are single determinations from individual experiments.

May et al. Fig S12

Gating for cells:

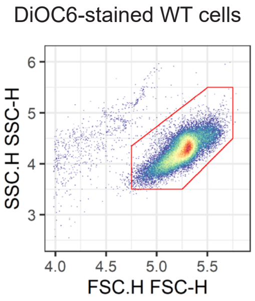

Gating for cells with high $\Delta\Psi_m$:

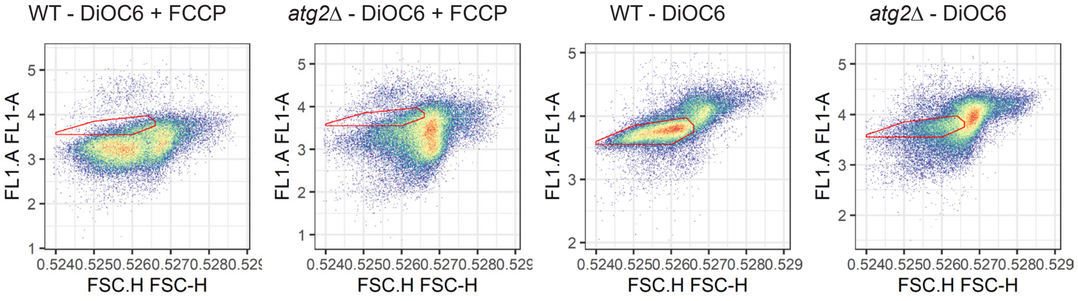

Supplementary Figure S12. Gating strategy used to determine cells with high mitochondrial membrane potential ($\Delta\Psi_m$). First, non-cell debris were removed by gating for cells by forward and side scatter. Following this, the proportion of highly fluorescent newly-emerged cells was identified by gating for the indicated region in the FL1-A channel. The proportion of cells falling within this region are shown in Fig. 5b. The logicle transformation was used for forward and side scatter data. The data shown in this figure are from the t = 26 h time point of the experiment shown in Fig. 5.

SUPPLEMENTARY INFORMATION

Supplementary Table 1: Antibodies used in this study

Resource	Supplier	Reference number
Mouse monoclonal Anti-GFP, clones 7.1 and 13.1	Roche	11814460001
Rabbit polyclonal Anti-Ape1	Ohsumi lab stock	NA
Mouse monoclonal Anti-β-actin	Wako	011-24554
Rabbit polyclonal Anti-Atg13	Ohsumi lab stock	NA

Supplementary Table 2: Reagents used in this study

Reagent	Supplier	Reference number
Difco Yeast Nitrogen Base w/o Amino Acids and Ammonium Sulfate	BD	233520
Ammonium sulfate	Nacalai	02620-04
Bacto Casamino acids	BD	223050
Bacto Yeast extract	BD	212750
Bacto Peptone	BD	211677
Glucose	Nacalai	16806-54
Ethanol	Wako	14713-53
Galactose	Wako	075-00035
Sucrose	Wako	193-09545
Glycerol	Wako	075-00616
Sodium acetate	Wako	190-01075
Raffinose	Wako	184-00015
Lactate	Wako	195-02305
L-Glutamic Acid	Wako	070-00502
L(+)-Glutamine	Wako	074-00522
Glycine	Wako	073-00732
L-Serine	Wako	199-00402
D-Serine	Wako	191-08821
Adecanol	Adeka	LG-109
FCCP	Sigma	C2920
Urea	Sigma	U6504
TRIzol	Life Tech	15596026
Methyl blue	Sigma	M6900
DiOC6	Molecular probes	D273
Triphenyltetrazolium chloride	Sigma	T8877
Adenosine 5'-triphosphate, [γ-^{32}P]-	PerkinElmer	NEG502Z
Met-^{35}S label (stabilized)	ARC Inc.	ARS0110A
T4 Polynucleotide Kinase	Toyobo	PNK-111
KOD -plus- Ver. 2	Toyobo	KOD-211
PerfectHyb Plus	Sigma	H7033

Supplementary Table 3: Strains used in this study

Strain	Source	Reference number
S. cerevisiae X2180-1B (*MATα SUC2 mal mel gal2 CUP1)*	Lab stock	ATCC#204505
S. cerevisiae X2180-1B *atg1Δ* (*MATα SUC2 mal mel gal2 CUP1 atg1Δ::KANMX)*	This study	yAM150
S. cerevisiae X2180-1B *atg2Δ* (*MATα SUC2 mal mel gal2 CUP1 atg1Δ::KANMX)*	This study	yAM151
S. cerevisiae X2180-1B *atg3Δ* (*MATα SUC2 mal mel gal2 CUP1 atg1Δ::KANMX)*	This study	yAM306
S. cerevisiae X2180-1B *atg4Δ* (*MATα SUC2 mal mel gal2 CUP1 atg1Δ::KANMX)*	This study	yAM308
S. cerevisiae X2180-1B *atg5Δ* (*MATα SUC2 mal mel gal2 CUP1 atg1Δ::KANMX)*	This study	yAM310
S. cerevisiae X2180-1B *atg6Δ* (*MATα SUC2 mal mel gal2 CUP1 atg1Δ::KANMX)*	This study	yAM312
S. cerevisiae X2180-1B *atg7Δ* (*MATα SUC2 mal mel gal2 CUP1 atg1Δ::KANMX)*	This study	yAM314
S. cerevisiae X2180-1B *atg8Δ* (*MATα SUC2 mal mel gal2 CUP1 atg1Δ::KANMX)*	This study	yAM316
S. cerevisiae X2180-1B *atg9Δ* (*MATα SUC2 mal mel gal2 CUP1 atg1Δ::KANMX)*	This study	yAM443
S. cerevisiae X2180-1B *atg10Δ* (*MATα SUC2 mal mel gal2 CUP1 atg1Δ::KANMX)*	This study	yAM318
S. cerevisiae X2180-1B *atg11Δ* (*MATα SUC2 mal mel gal2 CUP1 atg1Δ::KANMX)*	This study	yAM153
S. cerevisiae X2180-1B *atg12Δ* (*MATα SUC2 mal mel gal2 CUP1 atg1Δ::KANMX)*	This study	yAM320
S. cerevisiae X2180-1B *atg13Δ* (*MATα SUC2 mal mel gal2 CUP1 atg1Δ::KANMX)*	This study	yAM322
S. cerevisiae X2180-1B *atg14Δ* (*MATα SUC2 mal mel gal2 CUP1 atg1Δ::KANMX)*	This study	yAM340
S. cerevisiae X2180-1B *atg15Δ* (*MATα SUC2 mal mel gal2 CUP1 atg1Δ::KANMX)*	This study	yAM342

S. cerevisiae X2180-1B *atg16*Δ (*MAT*α *SUC2 mal mel gal2 CUP1 atg1*Δ*::KANMX)*	This study	yAM344
S. cerevisiae X2180-1B *atg17*Δ (*MAT*α *SUC2 mal mel gal2 CUP1 atg1*Δ*::KANMX)*	This study	yAM346
S. cerevisiae X2180-1B *atg18*Δ (*MAT*α *SUC2 mal mel gal2 CUP1 atg1*Δ*::KANMX)*	This study	yAM348
S. cerevisiae X2180-1B *atg19*Δ (*MAT*α *SUC2 mal mel gal2 CUP1 atg1*Δ*::KANMX)*	This study	yAM350
S. cerevisiae X2180-1B *atg20*Δ (*MAT*α *SUC2 mal mel gal2 CUP1 atg1*Δ*::KANMX)*	This study	yAM352
S. cerevisiae X2180-1B *atg22*Δ (*MAT*α *SUC2 mal mel gal2 CUP1 atg1*Δ*::KANMX)*	This study	yAM358
S. cerevisiae X2180-1B *atg23*Δ (*MAT*α *SUC2 mal mel gal2 CUP1 atg1*Δ*::KANMX)*	This study	yAM356
S. cerevisiae X2180-1B *atg24*Δ (*MAT*α *SUC2 mal mel gal2 CUP1 atg1*Δ*::KANMX)*	This study	yAM360
S. cerevisiae X2180-1B *atg27*Δ (*MAT*α *SUC2 mal mel gal2 CUP1 atg1*Δ*::KANMX)*	This study	yAM362
S. cerevisiae X2180-1B *atg29*Δ (*MAT*α *SUC2 mal mel gal2 CUP1 atg1*Δ*::KANMX)*	This study	yAM364
S. cerevisiae X2180-1B *atg31*Δ (*MAT*α *SUC2 mal mel gal2 CUP1 atg1*Δ*::KANMX)*	This study	yAM366
S. cerevisiae X2180-1B *atg32*Δ (*MAT*α *SUC2 mal mel gal2 CUP1 atg1*Δ*::KANMX)*	This study	yAM162
S. cerevisiae X2180-1B *atg33*Δ (*MAT*α *SUC2 mal mel gal2 CUP1 atg1*Δ*::KANMX)*	This study	yAM368
S. cerevisiae X2180-1B *atg34*Δ (*MAT*α *SUC2 mal mel gal2 CUP1 atg1*Δ*::KANMX)*	This study	yAM370
S. cerevisiae X2180-1B *atg36*Δ (*MAT*α *SUC2 mal mel gal2 CUP1 atg1*Δ*::KANMX)*	This study	yAM372
S. cerevisiae X2180-1B *atg38*Δ (*MAT*α *SUC2 mal mel gal2 CUP1 atg1*Δ*::KANMX)*	This study	yAM374
S. cerevisiae X2180-1B *atg39*Δ (*MAT*α *SUC2 mal mel gal2 CUP1 atg1*Δ*::KANMX)*	This study	yAM376

S. cerevisiae X2180-1B *atg40Δ* (*MATα SUC2 mal mel gal2 CUP1 atg1Δ::KANMX)*	This study	yAM378
S. cerevisiae X2180-1B *pep4Δ* (*MATα SUC2 mal mel gal2 CUP1 pep4Δ::KANMX)*	This study	yAM154
S. cerevisiae X2180-1B *fmt1Δ atg2Δ (MATα SUC2 mal mel gal2 CUP1 fmt1Δ::natNT2 atg2Δ::KANMX)*	This study	yAM237
S. cerevisiae X2180-1B *mis1Δ atg2Δ (MATα SUC2 mal mel gal2 CUP1 mis1Δ::natNT2 atg2Δ::KANMX)*	This study	yAM440
S. cerevisiae X2180-1B *mis1Δ (MATα SUC2 mal mel gal2 CUP1 mis1Δ::natNT2)*	This study	yAM461
S. cerevisiae X2180-1B *fmt1Δ (MATα SUC2 mal mel gal2 CUP1 fmt1Δ::natNT2)*	This study	yAM463
S. cerevisiae X2180-1B GFP-Atg8 (*MATα SUC2 mal mel gal2 CUP1 atg8*Δ::GFP - ATG8::hphNT1*)*	This study	yAM158
S. cerevisiae X2180-1B *atg2Δ* GFP-Atg8 (*MATα SUC2 mal mel gal2 CUP1 atg8*Δ::GFP - ATG8::hphNT1 *atg2Δ::KANMX)*	This study	yAM159
S. cerevisiae X2180-1B *atg11Δ* GFP-Atg8 (*MATα SUC2 mal mel gal2 CUP1 atg8*Δ::GFP - ATG8::hphNT1 *atg11Δ::KANMX))*	This study	yAM160
S. cerevisiae D273-10B (*MATα mal)*	Trevor Lithgow	yAM086
S. cerevisiae D273-10B *atg2Δ* (*MATα mal atg2Δ::KANMX)*	This study	yAM096
S. cerevisiae D273-10B *atg11Δ* (*MATα mal atg11Δ::KANMXMX)*	This study	yAM100

Supplementary Table 4: Plasmids used in this study

Name	Type	Source	Description
pFA6a-kanMX6	Plasmid	Janke et al.[51]	Generic plasmid for PCR-mediated generation of targeted deletion cassettes
pFA6a-natNT2	Plasmid	Janke et al.[51]	Generic plasmid for PCR-mediated generation of targeted deletion cassettes
pRS303-*GFP-ATG8*	Plasmid	Suzuki, et al.[50]	C-terminally tagged Atg8 for genomic integration following amplification of cassette by PCR.

Supplementary Table 5: Oligonucleotides used in this study

Name	Source	Sequence
ATG1_S1	This study	tttgaagctaccccatattttcaaatctcttttacaacaccagacgagaaattaagaaaatgCGTACGCTGCAGGTCGAC
ATG1_S2	This study	aagatacttgaaaatatagcaggtcatttgtacttaataagaaaaccatattatgcatcacATCGATGAATTCGAGCTCG
ATG1_8F	This study	taatataataaaaacataaggc
ATG1_5R	This study	caggtataaaagagctgg
ATG2_S1	This study	gcataaagattaaagcaaattaagaggaaccctttttttttttgatttcgatacaatgCGTACGCTGCAGGTCGAC
ATG2_S2	This study	cggccgaataattgccacaggtgcagctctagcaacataaactgctgcggcgctcggcccATCGATGAATTCGAGCTCG
ATG2_VerF-216	This study	cgcgataatgaatatgcaagg
ATG2_VerR+96	This study	cattttggctttcccacc
ATG3_S1	This study	tagaagttaggaacaaagaagtacaaaggagtaaatacaattttattatcCGTACGCTGCAGGTCGAC
ATG3_S2	This study	ctgtttttgaccacctggcttgcagctaatagtgaaaaaacacaaatttATCGATGAATTCGAGCTC
ATG3_2F	This study	tagtagatttcttattttc
ATG3_3R	This study	aatattcataatttatgtca
ATG4_S1	This study	ttagtagatgaagaatggacgacttcttatcacgtataggagtgatatacCGTACGCTGCAGGTCGAC
ATG4_S2	This study	gaatatattaaaacaagtatatatgcttatgaactagtgaattccttacaATCGATGAATTCGAGCTC
ATG4_VerF-218	This study	cacttcttccttctagaaactc
ATG4_VerR+244	This study	ggcattttggacttcgac
ATG5_S1	This study	ttgttcttttggttctagaagaacggagataggaaacctatgatgtaagtCGTACGCTGCAGGTCGAC
ATG5_S2	This study	tattttctgcgatatttgaatgacacttttaaatgcgtatataacagctcATCGATGAATTCGAGCTC
ATG5_2F	This study	atgatcgaagttttgtccgg
ATG5_3R	This study	aaagtatcaacagctctcga
ATG6_S1	This study	gccaaacatgtcacttacccctgcaactagacccatctttagaaggtcttagtCGTACGCTGCAGGTCGAC
ATG6_S2	This study	gtggaaaaagaaattttccctttatcacatttatgaaaaaatgcatttatatgaactacATCGATGAATTCGAGCTCG
ATG6_VerF-185	This study	ggactggtggtataatcgaa
ATG6_VerR+170	This study	ctggcatatcatctgatgaaag
ATG7_S1	This study	gctacttagataactaaagttcattatatttcaacaaatataagataatcaagaataaaatgCGTACGCTGCAGGTCGAC
ATG7_S2	This study	ggaaagtggcaccacaatatgtaccaatgctattatatgcaaaatattaagcaatctcatcATCGATGAATTCGAGCTCG
ATG7_VerF-196	This study	ggcaagcaagatttttcatttg
ATG7_VerR+261	This study	ggtagctttaccgacacttt
ATG8_S1	This study	tgataagagaatctaataattgtaaagttgagaaaatcataataaaaataattactagagacCGTACGCTGCAGGTCGAC
ATG8_S2	This study	cctataatttcgattttagatgttaacgcttcatttcttttcatataaaagactacctgccATCGATGAATTCGAGCTCG
ATG8_VerF-171	This study	gcacatgatatatcatcaccc

ATG8_VerR+207	This study	gctcacatttgtctccaaatac
ATG9_S1	This study	gcctgaaatatcaaaatcacggaattattaggttatggagagagatgaataccagt tacccCGTACGCTGCAGGTCGAC
ATG9_S2	This study	gccttatcttccgacgtcagacttcttgtaatactctttaacaagtcctaagacacca cccATCGATGAATTCGAGCTCG
ATG9_6F	This study	cccagtttcggaactttagggg
ATG9_5R	This study	attaggcttctcagagac
ATG10_S1	This study	gaagagaacaccatgaaaaaaaaaaaaaaagggctaaaaaacagaattatcag acttgatgCGTACGCTGCAGGTCGAC
ATG10_S2	This study	atatatatatatatttacatagatgattgcatagtgttttaaaaagctttcctaggttaag ATCGATGAATTCGAGCTCG
ATG10_VerF	This study	gcaatgagaagagaacacc
ATG10_VerR	This study	ccgacacatcatcagaaga
ATG11_S1	This study	attattttagtgtactgttgttgttcggaaagtacttcttttattttcttttatacatcatgC GTACGCTGCAGGTCGAC
ATG11_S2	This study	gttaaatagatacataattaaaatcttgtcatttgtgacaaacgtttagcactgttcaa acATCGATGAATTCGAGCTCG
ATG11_7F	This study	gcgatgcccttatttgagtacc
ATG11_6R	This study	ccttttccagagatgaggaaaattcaccagg
ATG12_S1	This study	aacgtacatccctaactgtatattctacagtagagtgaaccaatgacagtCGTA CGCTGCAGGTCGAC
ATG12_S2	This study	ttttgatcgactgtaggttttcttcttagaccattccagcgcccgggtatATCGA TGAATTCGAGCTC
ATG12_1F	This study	GTGATTTTCTTTTTCAGTGT
ATG12_2R	This study	TCAAACACTTTCGATGAAAT
ATG13_S1	This study	ccttccaggctcaagtcttgaaaagaaagcagaacatacagcccggttgaatagc atgagtcCGTACGCTGCAGGTCGAC
ATG13_S2	This study	gattatttttctttagttgtgccctttaaaataaaactttaccatttttaaccttctttagA TCGATGAATTCGAGCTCG
ATG13_VerF-150	This study	cgagaagtagaaacaagggt
ATG13_VerR+182	This study	gacacatggatcttcaagagg
ATG14_S1	This study	tagaaggataacgagtagagaaaaagggaagtaaaagttaaaaactagaatcct agtatgacCGTACGCTGCAGGTCGAC
ATG14_S2	This study	ctgactacatgcaactttatacacacggcaggaaaaaaagtgcgcactctagcct accacgATCGATGAATTCGAGCTCG
ATG14_VerF-237	This study	gcaatgccaatgatgaatatg
ATG14_VerR+70	This study	cactggctgctattgtttat
ATG15_S1	This study	atgttgcataaaagcccttcaagaaagagatttgcttctcctttgCGTACGCT GCAGGTCGAC
ATG15_S2	This study	ttacaactcgtatttggtgcagaagccaagccaattgcggcctacATCGATG AATTCGAGCTCG
ATG15_VerF-261	This study	gtgattgtgtgatcgcagt
ATG15_VerR+121	This study	ctcgtgtcgtttagctgaa
ATG16_S1	This study	ttgatttttgttattaacctgcgcgaatctgtattaagggcgcatattccCGTAC GCTGCAGGTCGAC

ATG16_S2	This study	gattttattttcttttgtatgcattttgtgacgatttgacaactgatgcaATCGATGAATTCGAGCTC
ATG16_VerF-177	This study	gctgcaactgtttatcctg
ATG16_VerR+143	This study	ggtgtactctctatagcatgt
ATG17_S1	This study	cgaagcagatgttacaaaatttgttaataatgccaggaaaacgctgaccgatgctCGTACGCTGCAGGTCGAC
ATG17_S2	This study	cgaaatattgatacaattattgaatctttgtaccgtatcctttttttcctttttttctaaggATCGATGAATTCGAGCTCG
ATG17_VerF-192	This study	cctctctcggttgtactattt
ATG17_VerR+268	This study	ccaacacagattcacagc
ATG18_S1	This study	cagttagtaatagtgttccagttaactctgtatccttttctcttcggcctgacaatgCGTACGCTGCAGGTCGAC
ATG18_S2	This study	gtgtatgcgttgtgacgtacggaaggcagcgcgagacacttccgtgatcaatccatcaagATCGATGAATTCGAGCTCG
ATG18_VerF-105	This study	ggtaactcagcagtggtc
ATG18_VerR+120	This study	gcaacaagataatggtgacg
ATG19_S1	This study	gaatcgaggtaattgcggcggcacttgcttcagtaacgcccaaaggagagttctggtaaatgCGTACGCTGCAGGTCGAC
ATG19_S2	This study	gtatgtgaaaaggtactcattgctgtataaaaatagagtttgacctagagttcttcccaagATCGATGAATTCGAGCTCG
ATG19_VerF-82	This study	ggtgtatcagacgaaagaaga
ATG19_VerR+140	This study	gctaggtgataagtaatgagagg
ATG20_S1	This study	cgaatactggtggtgcaattgaacataccatctagaaaagttgtcttctacagcacacaatgCGTACGCTGCAGGTCGAC
ATG20_S2	This study	acgttgtttatatatatatatatcaagtatgctataacgctaaaaaaaaatgctcataataATCGATGAATTCGAGCTCG
ATG20_VerF-245	This study	gctgtttcaatcttgctcatc
ATG20_VerR+273	This study	gcgatggagcataatcatct
ATG22_S1	This study	gcccttataagcaatagtttgctcataacatattctctacattagatatgCGTACGCTGCAGGTCGAC
ATG22_S2	This study	ccttatatatatacgtaagcaaagttttatgtaacaaaaaaaaaaaaaagaactaATCGATGAATTCGAGCTCG
ATG22_VerF-121	This study	cctcagctattgaaagatgc
ATG22_VerR+149	This study	cgtagttgcatcaaaccc
ATG23_S1	This study	ccacattttaacctcgttgttctataaggtaacaaaataaagtgaagaagtaaatatgCGTACGCTGCAGGTCGAC
ATG23_S2	This study	cgtgggcatacaatgtttaaatttacattatcctcatggctactctagctatttgcattATCGATGAATTCGAGCTCG

ATG23_VerF-297	This study	cctagccctaggacaact
ATG23_VerR+255	This study	gcctagatcatgatcttgaagt
ATG24_S1	This study	taagccgttattaaactttatttacggtataccacaatactgctctttttgttgaggatatgCGTACGCTGCAGGTCGAC
ATG24_S2	This study	atcagataatcgtagtgcccaaggtattatcagtagtaatgggaaaacattaagagcaccaATCGATGAATTCGAGCTCG
ATG24_VerF-95	This study	cgacgcaagattaacagattaaa
ATG24_VerR+207	This study	ggaagaacaatgcaaaatgttt
ATG27_S1	This study	gatttatatctgagatcttcaatcgatgcgatagataaaggtaaggaaagctttcacgatgCGTACGCTGCAGGTCGAC
ATG27_S2	This study	atataataaaaggttagcactgctgttgcaaaaatatcgaattgtaagccagtaaacttatATCGATGAATTCGAGCTCG
ATG27_VerF-159	This study	gtccaggtgcaaattgaaaa
ATG27_3R	This study	gacgaaactgttcttctgg
ATG29_S1	This study	cttatcaagaagagacatacataattgtactgctgtgcattttcctacttgactttcatgCGTACGCTGCAGGTCGAC
ATG29_S2	This study	ccaacgcagatttactaacacttaagctcgaagataaatcgtcatctgaactaccgcATCGATGAATTCGAGCTCG
ATG29_VerF-136	This study	cttcaagtacactatttctagacg
ATG29_VerR+249	This study	gcctcagacagcagagaata
ATG31_S1	This study	cgtcgcattcccattttcttattaagccggtaaacattgctgaaatctgcgaacaggaatgCGTACGCTGCAGGTCGAC
ATG31_S2	This study	cctacataaacaagttaagagagtctcatccatgcggcttcatttttgctttcatacggATCGATGAATTCGAGCTCG
ATG31_VerF-200	This study	ggtccagtggttcaaga
ATG31_3R	This study	gtcgatttggtagaaagggt
ATG32_S1	This study	gataagcaatattgaagtcctaatcacaaaagcaaaaaaaaatctgccaggaacagtaaacatCGTACGCTGCAGGTCGAC
ATG32_S2	This study	aacagaagtgatagtaaaaaagtgagtaggaacgtgtatgtttgtgtatattggaaaaaggATCGATGAATTCGAGCTCG
ATG32_1F	This study	ATTTAGGAAATAAGGTGCAG
ATG32_2R	This study	AATTTGCTAACAGAAGAAAT
ATG33_S1	This study	ttagccaaattcgcttatcacttactacttcacgatatacagaagtaaaaacatataaaaagCGTACGCTGCAGGTCGAC
ATG33_S2	This study	agaggagccgagttcaggcagctacaaattcttgtagagataaatacgtgaacgagaaacaATCGATGAATTCGAGCTCG
ATG33_VerF-95	This study	ggcaaaagaggtcccttg
ATG33_VerR+222	This study	gagcacctacaaatgtgctt
ATG34_S1	This study	ctagttcctataggttgagtgtctatcaaaaatttacggagacgcgatgCGTACGCTGCAGGTCGAC
ATG34_S2	This study	gttaaataagtactatagccaaagaaactggaagaatataaaaaagcatttaATCGATGAATTCGAGCTCG

ATG34_VerF-225	This study	ctgtgagcttgggtatacag
ATG34_VerR+148	This study	ctgtcttaggtttctcaacag
ATG36_S1	This study	gtattcagggcttaaaatactaaaatttggtggtcagtacagctcattaatgCGTACGCTGCAGGTCGAC
ATG36_S2	This study	gatggtgttcggacaacgttttagaatgagggtatctaactttcttctaATCGATGAATTCGAGCTCG
ATG36_VerF-162	This study	catcgttatacacgtacgatc
ATG36_VerR+221	This study	ctggagagcacacacaaa
ATG38_S1	This study	gttcgataattcaacgacagttatactaatcttggtgatggaatgCGTACGCTGCAGGTCGAC
ATG38_S2	This study	gttaaaagacgatacttcgtatcttgaaaagaaattaccttattcgtctaATCGATGAATTCGAGCTCG
ATG38_VerF-97	This study	gcgtaatggaacgctct
ATG38_VerR+247	This study	gcatccttgattccgtttc
ATG39_S1	This study	cgataatagagactagtaaaacagtcgagttgtcggacctaaaatgCGTACGCTGCAGGTCGAC
ATG39_S2	This study	cgttttttttttcttttgttaatttcattcttcatgctgggttttggatgatctaATCGATGAATTCGAGCTCG
ATG39_VerF-245	This study	caaggtacctggatcgag
ATG39_VerR+247	This study	gagatggatagcatacgttatg
ATG40_S1	This study	cctaacgttctttctgctgtgcttcactccaccatagaaaactaatgCGTACGCTGCAGGTCGAC
ATG40_S2	This study	ggtaccttcatagactaccattatggtaaaatggaaaaactattctaATCGATGAATTCGAGCTCG
ATG40_VerF-182	This study	cctaagcagcgaaatacac
ATG40_VerR+201	This study	ccgatacagcatgtttaacc
PEP4_S1	This study	agcctagtgacctagtatttaatccaaataaaattcaaacaaaaaccaaaactaacatgttcCGTACGCTGCAGGTCGAC
PEP4_S2	This study	ctagatggcagaaaaggatagggcggagaagtaagaaaagtttagctcaaattgctttggcATCGATGAATTCGAGCTCG
PEP4_1F	This study	gggaagaataacaaaaagtatatctcaccc
PEP4_2R	This study	cgggctacccgcatataatgacattatggg
FMT1_S1	This study	ggggacctaggcacctcatagtgagaagattgaaaacggcgtatcaaacaaatgCGTACGCTGCAGGTCGAC
FMT1_S2	This study	gaaagaagggagaagaagaatactaaaagaggaatgtagagccgggttaATCGATGAATTCGAGCTCG
FMT1_VerF	This study	gctcatctcggtttcttaat
FMT1_VerR	This study	gggcttcctacacttaattca
MIS1_S1	This study	tctcagtctgttcatttgtcagatatttaaggctaaaaggaaatgCGTACGCTGCAGGTCGAC

MIS1_S2	This study	catcgaggtcgaattgatgccattaggagtctgccaaactatttaATCGATGAATTCGAGCTCG
MIS1_VerF	This study	ctcctgcaacactcttctat
MIS1_VerR	This study	gctttattgaaggaagacgc
Mitochondrial initiator tRNA oligonucleotide probe for northern blotting	Li, et al.[29]	tagcaataatacgatttg
